# Enhancing flowering plant functional richness improves wild bee diversity in vineyard inter‐rows in different floral kingdoms

**DOI:** 10.1002/ece3.7623

**Published:** 2021-05-04

**Authors:** Sophie Kratschmer, Bärbel Pachinger, René Gaigher, James S. Pryke, Julia van Schalkwyk, Michael J. Samways, Annalie Melin, Temitope Kehinde, Johann G. Zaller, Silvia Winter

**Affiliations:** ^1^ Institute of Zoology University of Natural Resources and Life Science (BOKU) Vienna Austria; ^2^ Institute for Integrative Nature Conservation Research University of Natural Resources and Life Science (BOKU) Vienna Austria; ^3^ Department Conservation Ecology and Entomology Stellenbosch University Stellenbosch South Africa; ^4^ Compton Herbarium South African National Biodiversity Institute Cape Town South Africa; ^5^ African Centre for Coastal Palaeoscience Nelson Mandela Metropolitan University Port Elizabeth South Africa; ^6^ Department of Zoology Obafemi Awolowo University Ile‐Ife Nigeria; ^7^ Institute of Plant Protection University of Natural Resources and Life Science (BOKU) Vienna Austria

**Keywords:** Apiformes, country comparison, floral traits, functional traits, management intensities, viticultural landscapes

## Abstract

Wild bees are threatened by multiple interacting stressors, such as habitat loss, land use change, parasites, and pathogens. However, vineyards with vegetated inter‐rows can offer high floral resources within viticultural landscapes and provide foraging and nesting habitats for wild bees. Here, we assess how vineyard management regimes (organic vs. conventional; inter‐row vegetation management) and landscape composition determine the inter‐row plant and wild bee assemblages, as well as how these variables relate to functional traits in 24 Austrian and 10 South African vineyards. Vineyards had either permanent vegetation cover in untilled inter‐rows or temporary vegetation cover in infrequently tilled inter‐rows. Proportion of seminatural habitats (e.g., fallows, grassland, field margins) and woody structures (e.g., woodlots, single trees, tree rows) were used as proxies for landscape composition and mapped within 500‐m radius around the study vineyards. Organic vineyard management increased functional richness (FRic) of wild bees and flowering plants, with woody structures marginally increasing species richness and FRic of wild bees. Wild bee and floral traits were differently associated across the countries. In Austria, several bee traits (e.g., lecty, pollen collection type, proboscis length) were associated with flower color and symmetry, while in South African vineyards, only bees’ proboscis length was positively correlated with floral traits characteristic of Asteraceae flowers (e.g., ray–disk morphology, yellow colors). Solitary bee species in Austria benefitted from infrequent tillage, while ground nesting species preferred inter‐rows with undisturbed soils. Higher proportions of woody structures in surrounding landscapes resulted in less solitary and corbiculate bees in Austria, but more aboveground nesting species in South Africa. In both countries, associations between FRic of wild bees and flowering plants were positive both in organic and in conventional vineyards. We recommend the use of diverse cover crop seed mixtures to enhance plant flowering diversity in inter‐rows, to increase wild bee richness in viticultural landscapes.

## Graphical abstract

This article studies how wild bee and plant diversity and functional traits are related to vineyard management intensities and viticulture landscape properties in Austria and South Africa. Common denominators for wild bees and flowering plants are studied across countries. Unique characteristics of the bee and plant communities in the vineyards of the two countries are analyzed using functional traits to provide subtlety conservation measures for wild bees in viticultural agroecosystems.

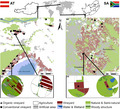

## 1 INTRODUCTION

Agricultural intensification drives plant species declines (Beckmann et al., 2019), leading to simplified communities with reduced ecosystem stability and resilience (Tilman et al., 2014). Similar trends are reported for insects (Samways, 2020), specifically insect pollinators (Potts et al., 2010), threatening agroecosystem function, and human food security (Vanbergen et al., 2013). This has impacted maintenance of floral diversity in the wider landscape, as over 85% of wild flowering plants depend on animal pollination (Ollerton et al., 2011). The interaction between plants and insect pollinators increases the risk of cascading extinctions, especially due to land use change, which ultimately leads to the depletion of ecosystem function (Papanikolaou et al., 2017; Weiner et al., 2014). Bee species that are specialized pollinators of particular plants or habitats are highly vulnerable to land use change compared with generalist species, leading to a decrease in plants they pollinate (Biesmeijer et al., 2006).

Worldwide, about 7.4 million ha of land is cultivated as vineyards (OIV, 2019). Vineyards are often intensively managed perennial monocultures, with high pesticide application rates (Urruty et al., 2016), which greatly affect nontarget species. Moreover “weeds” are frequently eradicated in vineyards to reduce potential competition for water and nutrients (Gago et al., 2007; Pardini et al., 2002; Zaller et al., 2018). However, vineyards that are managed ecologically sensitively can offer pollinator‐friendly areas that conserve biodiversity (Cox & Underwood, 2011; Viers et al., 2013) and promote ecosystem services (James et al., 2015; Wratten et al., 2012). The complex vegetation structure in perennial crops, such as vineyards or fruit orchards (Carvalheiro et al., 2012), increases their potential to host diverse plant and arthropod communities in the inter‐row space between vines or trees (Bruggisser et al., 2010). Although grapevines are not dependent on insect pollination, the inter‐rows can provide important floral resources for insect pollinators (Kehinde & Samways, 2014a; Kratschmer et al., 2019) or parasitoids (Danne et al., 2010; Judt et al., 2019), contributing to ecosystem services such as pollination or pest control (Danne et al., 2010; Shields et al., 2016; Winkler et al., 2017). At the landscape scale, viticultural agroecosystems are often composed of seminatural habitats (SNHs) such as single trees, dry grasslands, or hedges (Boller et al., 1997; Eichhorn et al., 2006). These landscape elements can provide additional habitat and food sources for natural enemies (Corbett & Rosenheim, 1996) and pollinators (Gillespie & Wratten, 2012; Kratschmer et al., 2018). Agricultural landscapes that include a high proportion of natural or seminatural habitat potentially offset the negative impacts of intensive agricultural management on biodiversity (Kohler et al., 2008), pollination, or pest control resulting in reduced insecticide use (Paredes et al., 2020).

Wild bees are efficient pollinators of both crops and wild plants (Klein et al., 2007; Mallinger & Gratton, 2015; Ollerton et al., 2011), due to trait matching between plant and bee taxa. For instance, long‐tongued bees (Megachilidae and Apidae) use their elongated glossa to access the nectar from long‐tube corolla flowers (Krenn et al., 2019; Michener & Brooks, 1984). Studying responses of general metrics such as abundance or species richness along with functional trait metrics that capture the community structure will improve conservation measures for wild bees (Vereecken et al., 2020). Functional richness (FRic) measures the amount of niche space occupied by various species within a community. Depending on the traits used, it measures niche complementarity or resilience of a community against environmental disturbance (Mason et al., 2005). Pollination efficiency benefits from high FRic of bees, as increased niche complementarity by different traits allows plants with multiple floral traits to be pollinated (Junker et al., 2013).

Austrian wild bee diversity compared with other Central European countries is very high with 702 species documented (Wiesbauer, 2020), which is related to the high diversity of habitats (alpine to low‐land) and climatic regions within a small area. The Cape Floristic Region (CFR) has exceptionally high biodiversity with an extremely high proportion of endemic bee species (Kuhlmann, 2009) compared with similar Mediterranean‐type ecosystems in other regions (Valente & Vargas, 2013). To date, 941 bee species have been described in South Africa (Eardley & Coetzer, 2016; Eardley & Urban, 2010; Melin & Colville, 2019), but about 27% of genera have yet to be revised. The Cape Floristic Region with a size of about 90,000 km^2^ comprises about 9,000 vascular plant species with 69% endemic to the region (Goldblatt & Manning, 2002). This influences the species pool of bees and plants that interact within agricultural and other human‐impacted landscapes (Linder et al., 2010). Austria is similar in size (84,000 km^2^) but comprises only 2,950 vascular plants with 5% endemic species (Rabitsch & Essl, 2008). Biodiversity hot spots such as Austria and South Africa are critical for biodiversity conservation (Myers et al., 2000; Habel et al., 2013; Tiefenbach et al., 2014) and are threatened by land use change. In the CFR, the highly diverse natural habitats fynbos and renosterveld are threatened with conversion to vineyards with increases in vineyard area from 1994 to 2015 by about 30% (Fairbanks et al., 2004; OIV, 2019), although decreased since then by about 10% in 2018 (OIV, 2019). In contrast, the vineyard area in Austria decreased by about 35% from a maximum in the 1980s in relation to 2015 (ÖWM, 2020). However, small landscape elements such as single trees are still removed to facilitate machinery use in vineyards and other crops. Thus, understanding the drivers of functional richness of pollinators and plants at the vineyard and landscape scale would highlight what is important for pollinator conservation in these regions and beyond.

The focus of this study is on wild bee assemblages in vineyards across two floral kingdoms (in Austria and South Africa) with their different landscapes, histories, and climates to evaluate the influence of common versus unique drivers of diversity in vineyards. Firstly, we hypothesized that wild bee species and FRic are positively affected by less intensive vineyard management and high landscape diversity. This is because complex landscapes have been shown to mitigate the negative effects of intensive vineyard management and low functional flowering plant richness on wild bees. Secondly, we predict that flowering plant FRic is predominantly influenced by vineyard management, while landscape composition plays a minor role. Thirdly, due to the unique bee and plant communities evolved in the two study regions, specific wild bee and floral traits are hypothesized to show different association patterns, though in accordance with trait matching. Finally, trait association patterns reflect vineyard management practices and different landscape features of the two countries.

## 2 METHODS

### 2.1 Study sites

Wild bees, flowering insect‐pollinated plants (“flowering plants” from here onwards) in the vineyard inter‐rows, farm management (organic vs. conventional), inter‐row vegetation cover (as proxy for vegetation management intensity), and landscape composition were assessed in two viticultural regions in Austria and South Africa. Austria is located in the Palearctic biome and the Holarctic Floral Kingdom, whereas the study sites in South Africa are located in the Fynbos biome of the Cape Floristic Region, a Mediterranean‐type ecosystem that supports expansive species radiation and endemism among indigenous plants (Johnson et al., 2006).

**FIGURE 1:**
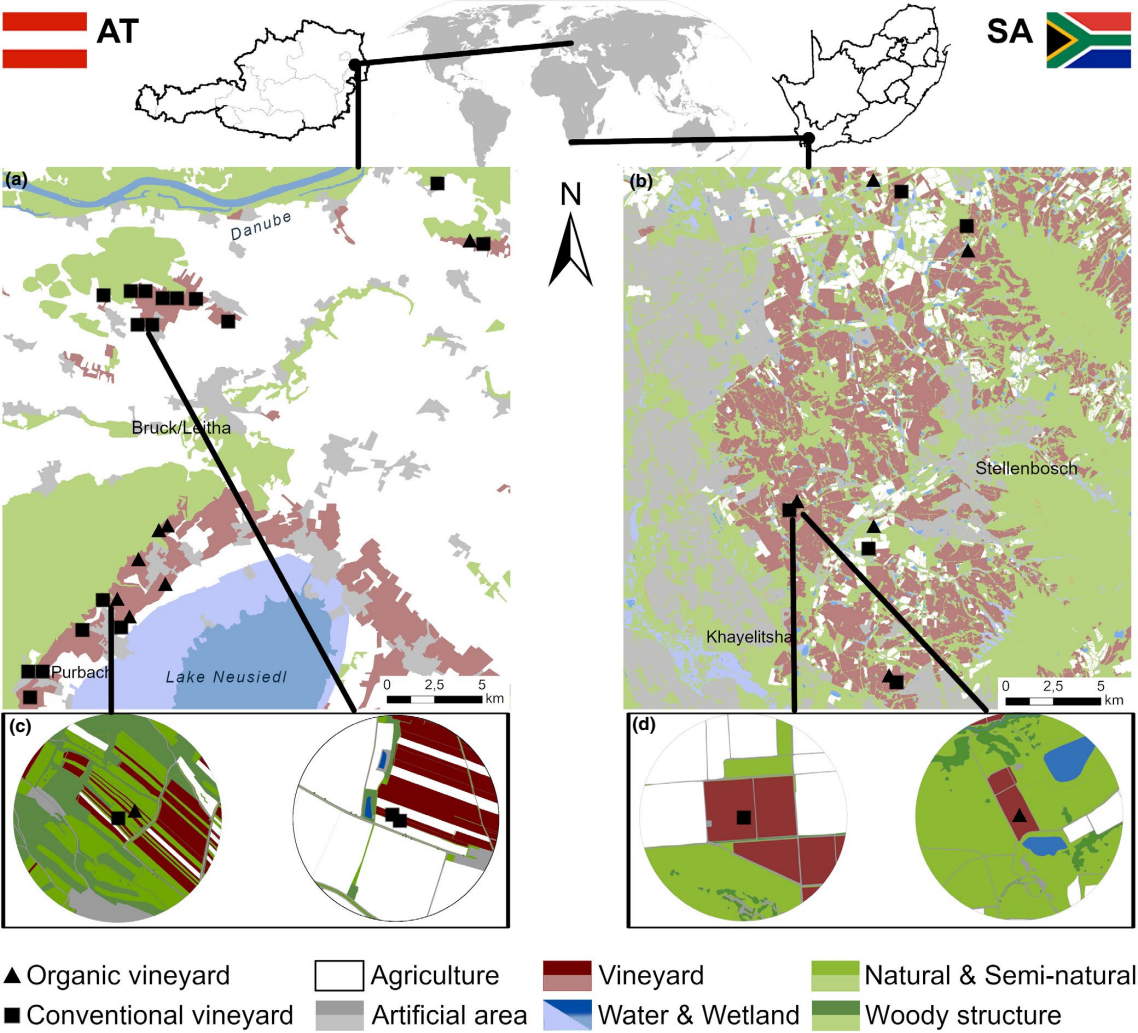
Study regions and localities of study vineyards in (a) Austria and (b) South Africa including respective farm type and landscape properties according to CORINE land cover (Umweltbundesamt GmbH, 2012) and DEA/CARDNO (GEOTERRAIMAGE, 2015). Detailed examples of landscape buffers (500 m) with a relatively high abundance of natural/seminatural habitats and high cover of agriculture for (c) Austria and (d) South Africa. Note Legend: Light shadings refer to maps of study regions (a, b), and darker colors refer to landscape circles (c, d)

Austrian study vineyards were located in two Eastern Austrian viticultural areas (Carnuntum: 48°04′N, 16°47′E and Neusiedlersee–Hügelland: 47°54′N, 16°41′E) characterized by rain‐fed vineyards consisting of small parcels (0.4–1.0 ha) with trellis systems on plain or hilly terrain. The study area is characterized by a small‐scaled agricultural landscape with vineyards, arable fields, seminatural habitats (SNHs), woods, and villages (Figure 1a) (Kratschmer et al., 2018). The average distance between sites was 13.4 km. The climate is classified as warm temperate (Cfb according to the Köppen–Geiger climate classification (Kottek et al., 2006)). During the two study years (2015 and 2016), average air temperature was 11.5°C and 11.1°C, and annual precipitation was 508 and 636 mm, respectively (ZAMG, 2017). In Austria, 24 selected vineyard inter‐rows were either covered with permanent vegetation (no tillage for >5 years) or temporary vegetation (alternating tillage in every second inter‐row) in the center of 16 landscape buffers (Figure 1a,c). In eight of these landscape buffers, paired vineyards differing in inter‐row vegetation management regimes (*n* = 16) were studied (Figure 1a). Management information was gathered by means of personal interviews with the winegrowers (Table 1). Conventional vineyard management used herbicides and mechanical weed control only under grapevines, and additional fungicides. Organic winegrowers only used mechanical weed control and copper and sulfur for fungal control. No synthetic insecticides were used in the study vineyards six years prior to the study. The inter‐rows were covered with either seeded cover crops or spontaneous vegetation from the existing seed bank or surrounding vegetation (Table 1).

**TABLE 1:** Characteristics of vineyard and landscape‐scale variables in the two studied wine‐growing regions in Austria and South Africa

Variables	Austria	South Africa
Vegetation cover in the inter‐rows (% mean ± *SD*)	81.95 ± 10.48	51.16 ± 29.00
Inter‐row vegetation management		
Method	No tillage and alternating tillage	Tillage
Number of tillage operations per year	0–3	1–3
Number of vineyards with seeded cover crops	18	10
Number of vineyards with spontaneous vegetation only	6	0
Farm type		
Number of organic vineyards	7	5
Number of conventional vineyards	17	5
Landscape		
Number of buffers	16	10
SNH cover (% mean ± *SD*)	19.85 ± 14.27	28.25 ± 21.76
Woody structure cover (% mean ± *SD*)	15.45 ± 17.94	5.50 ± 1.80
Vineyard cover (% mean ± *SD*)	32.81 ± 18.00	47.11 ± 27.28

Abbreviation: SNH, Seminatural habitat excluding woody structures.

The South African study vineyards were located in the Western Cape Province (33°57’S, 18°46’E) near the town of Stellenbosch, which are characterized by large rain‐fed vineyards (4–10 ha size) located on plain or hilly terrain, with natural habitats (i.e., fynbos and renosterveld vegetation) surrounding the vineyards. However, there were also high‐density patches of invasive alien tree species (mostly *Pinus* spp., *Eucalyptus* spp., and *Acacia* spp.) and deciduous fruit and olive orchards (Figure 1b). The climate is Mediterranean‐type (Csb according to the Köppen–Geiger classification (Kottek et al., 2006)), the mean annual temperature was 17.9°C and 16.6°C, and annual precipitation was 600.2 and 463.9 mm, respectively (Meijers, 2020) for the two years of investigation (2009 and 2010). Five pairs of organic and conventional vineyards, each within 0.13–1 km distance (Figure 1), were surveyed. The average distance between sites was 12.9 km. The guidelines for conventional and organic vineyard management are similar to those described above, but conventional winegrowers in South Africa use low‐risk insecticides sparingly as part of the Integrated Production of Wine scheme of SA (Wine and Spirit Board, 2020). The inter‐rows of both organic and conventional vineyards were covered with vegetation (Table 1) seeded with cover crops such as *Hypochaeris radicata, Raphanus raphanistrum, Erodium moschatum, Bidens pilosa, Avena fatua,* and *Vicia* spp. (Kehinde & Samways, 2012), as well as species emerging from the soil seed bank.

### 2.2 Sampling designs for wild bees and flowering plants

**FIGURE 2:**
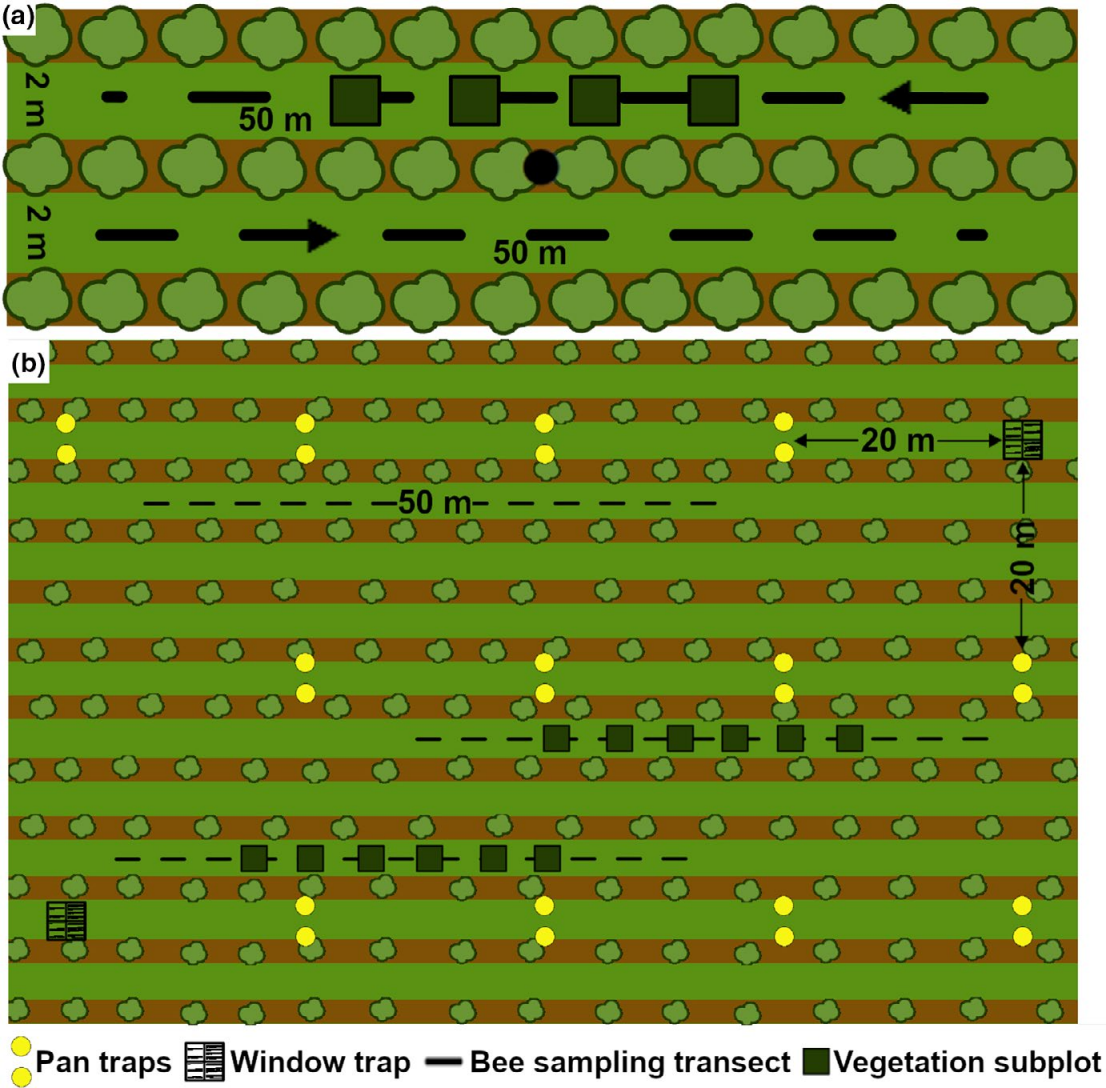
Overview of wild bees, flowering plants, and vegetation cover sampling design in (a) Austrian and (b) South African vineyards

In Austria, wild bees were sampled using 100‐ to 130‐m‐long transects (transect width given by inter‐row width) in two neighboring inter‐rows per vineyard (Figure 2a). Transect length was adapted according to the width of the inter‐row, which ranged between 1.5 and 2 m. Sampling was conducted monthly between April and August, resulting in 5 transect walks in every vineyard in both study years. Each sampling was done within 2–3 days between 9 a.m. and 4 p.m. on sunny, less windy days with temperatures above 15°C and dry vegetation. To avoid time of day bias, each vineyard was visited at different times of the day throughout the sampling period. During a sampling period of 15 min per transect, all bees observed were collected with a handheld insect net for later identification in the laboratory. Honey bees and most bumblebee species were identified and counted in the field. The five sampling dates per study year were adjusted to the vine's phenological stages, also complying with wild bee sampling recommendations (Schindler et al., 2013), starting in April (first leaf buds) until September (start of grape maturation). Bees were identified to species level based on identification keys (Schmid‐Egger & Scheuchl, 1997; Amiet et al., 1999, 2001, 2004, 2007, 2010; Scheuchl, 2000, 2006; Gokcezade et al., 2010; Dathe et al., 2016) using the nomenclature according to Gusenleitner et al. (2012). Voucher specimens were deposited at the Institute for Integrative Nature Conservation Research (INF) at BOKU (Vienna). Number and cover of flowering plants were also recorded along each sampling transect and identified to species using Fischer et al. (2008). Vegetation cover (%) and plant species richness including non‐insect‐pollinated plants (Hall et al., 2020) were estimated twice per study year (at the beginning of the vegetation period and 2 months later) in four 1 × 1 m subplots of one inter‐row per vineyard.

The South African wild bee data represent a combination of three sampling methods performed in each vineyard in spring to summer (August to December) 2009 and 2010 (Figure 2b). In 2009, 12 yellow (non‐UV) pan traps (capacity of 1,000 ml/trap) and two window intercept traps (0.5 × 0.5 m) were left in the field for five consecutive days on two sampling dates. Pan and window traps were arranged in pairs (traps in a pair were 2 m apart) with a distance of 20 m between each site and from the edge of the field. In 2010, sampling was performed by walking the transect, collecting wild bees with an aerial net in a predefined plot (100 × 50 m) per vineyard. Within these plots, three 50 × 2 m transects were placed randomly and sampled six times with a two‐week interval between visits, with each transect sampled for 20 min resulting in 1 hr of sampling per plot (Kehinde & Samways, 2012, 2014a, 2014b). Transect walks were done on days without rain, minimal wind, minimal cloud (< 5%), temperature >15°C, and between 9 a.m. and 4 p.m. Bees were identified to species or morphospecies (especially Halictidae) (Michener, 2000). Voucher specimens were deposited at the National Collection of Insects, Pretoria, South Africa. Flowering plant species data were collected in 2010 during the plant–pollinator interaction field survey (Kehinde & Samways, 2014c) and considered to be similar to 2009 as management remained constant (Lososová et al., 2003). Both vegetation cover (%) and total plant species richness were assessed in 2009 along two transects per vineyard plot. Each transect consisted of six 2 × 2 m subplots with 5‐m intervals located in the vineyard center (Kehinde & Samways, 2012).

Each bee species was described in terms of 7 functional traits (Table 2) that are recognized as important for bee autecology (Michener, 2007). All but two of the traits were obtained from relevant literature (Greenleaf et al., 2007; Scheuchl & Willner, 2016; Westrich, 2018) and expert evaluation (for the South African species, inferences were made based on available literature (Eardley, 2013; Eardley & Urban, 2010; Gess & Gess, 2014; Kuhlmann et al., 2011; Michener, 2007). Lecty and sociality were only used for the Austrian bee data, because ecological information on the South African bees was limited. Further, species of the genera *Lasioglossum* and *Halictus* vary greatly in their sociality; thus, inferences based at the genus level for bees identified to morphospecies were not accurate. For lecty or the degree of pollen specialization, we assigned two categories: polylecty and oligolecty. According to Cane and Sipes (2006), polylecty includes broad polylecty, polylecty, and mesolecty, whereas oligolecty joins narrow oligolecty and monolecty. The foraging range of wild bees has been shown to increase with body size, a standard measure for body size is intertegular distance (ITD) or the distance between the tegulae (Greenleaf et al., 2007). We measured the ITD (Cane, 1987; Greenleaf et al., 2007) of 1–5 individuals per bee species from Austria using a Keyence VHX‐5000 digital microscope and 9–24 individuals per bee species from South Africa using a Leica Z16 APO stereoscope. Austrian bumblebees were identified in the field; thus, we measured the ITD from five specimens per species selected from the collection at INF, BOKU (Vienna), sampled in eastern Austria. Mouthpart length (i.e., length of proboscis of each bee species was estimated based on bee family and average ITD (Table S1) using the R package *BeeIT* (Cariveau et al., 2016). As Melittidae bees were not included in this package, the species’ mouthpart length was estimated using the parameter values of the allometric power function as reported by Melin et al. (2019) and revised using the R package *pollimetry* (Kendall, 2018).

**TABLE 2:** Wild bee and flowering plant traits used for calculation of functional trait richness and community‐weighted means. Sociality and Lecty only used for Austrian bees due to lack of information from South Africa

Trait	Description and trait categories (in *italics*)
Wild bees	
Sociality	Females of s*olitary* species nest and breed alone.@@*Eusocial* bees divide tasks (egg‐laying, foraging) between castes.
Nesting	*Ground nesting* species excavate nests in the ground.@@*Aboveground nesters* require preexisting cavities, dead wood, or plant stems.
Lecty	*Polylectic* bees collect pollen from different plant taxa (i.e., broad polylecty, polylecty, mesolecty cf. Cane & Sipes, 2006)@@*Oligolectic* bees are specialized on closely related/single plant taxa. (i.e., narrow oligolecty, monolecty cf. Cane & Sipes, 2006)
Body size	*ITD* (mm): shortest linear distance measured between wing tegulae across the dorsal thorax (Cane, 1987).
Mouthpart length	*Proboscis* (mm): Sum of prementum and glossa length estimated from ITD and family using *Bee IT* and *pollimetry* (Cariveau et al., 2016; Kendall, 2018; Melin et al., 2019).
Pollen collection type	Part of the body where pollen is stored for transport: *Corbicula; tibial scopa;* a*bdominal scopa; crop* (ingested).
Seasonality	Activity during vegetation period: *Spring* (AT: III – V, SA: IX – XI); *Early summer* (AT: V – VI, SA: XI – XII); *Summer* (AT: VI – VIII, SA: XII – II); *Late summer* (AT: IX, SA: III); *Whole vegetation period* (AT: III – X, SA: IX – IV).
Flowering plants	
Flower morphology (Kugler, 1970)	Flowers classified according to shape: *bell and funnel flowers*; *disk flowers*; *flag blossom*; Asteraceae, *only disk flower heads*; Asteraceae, *only ray flower heads*; Asteraceae, *ray and disk flower heads*; *lip flowers*; *wind‐pollinated and pollen flowers*.
Nectar accessibility (Müller, 1881)	Classification according to location of reward: *Bee flower*; *Fabaceae type*; *nectar* *±hidden*; *flower associations with totally hidden nectar*; *flowers with totally hidden nectar*; *nectar openly available*; *wind‐pollinated and pollen flowers*.
Nectar presence	Flowers with *nectar present* or *no nectar present*.
Flower symmetry	Flowers with *radial* or *bilateral* symmetry.
Flower color	Different shades of the following colors were grouped: *blue*; *pink*; *purple*; *red and orange*; *white*; *white* *and yellow*; *yellow*
Seasonality	As above

Each flowering plant species was described by 6 functional traits (Table 2) selected according to the relevance for pollination, flower ecology, and morphology (e.g., accessibility of reward, flower morphology, or flower color). They were obtained from TRY plant trait database, version 5, released in March 2019 (www.try‐db.org; Kattge et al., 2020; see full list of references in Appendix—TRY References). Additionally, information on flower morphology was extracted from Kugler (1970), and seasonality from Fischer et al. (2008) for the Austrian taxa, and from Manning and Goldblatt (2012) for South Africa. The information derived from the TRY database and other literature was nominally scaled, but due to their great detail, these were not suitable for our statistical analysis; therefore, they were categorized prior to data analysis (Table S2).

### 2.3 Landscape evaluation

Landscape composition in both countries was evaluated within a 500 m buffer around each vineyard, as this radius covers the flight range of most wild bee species (Zurbuchen et al., 2010; Zurbuchen & Müller, 2012). In Austria, field mapping was performed in July 2015 following the EUNIS habitat‐type classification (European Environment Agency, 2016), using the Austrian land utilization map (BMLFUW, 2012). The digitizing of field data and calculation of the proportions of different landscape features (Table 3) was done in ArcGIS (ESRI, 2013). For South Africa, landscape mapping was based on high‐resolution (50 cm) orthorectified aerial photographs taken in 2008 and 2010 (NGI, 2020) and digitizing done in QGIS v3.12.1 (QGIS Development Team, 2020). The habitat‐type classification used in AT was adapted to account for the South African landscape characteristics (Table 3).

**TABLE 3:** Landscape classification based on mapped landscape entities according to EUNIS habitat classification (European Environment Agency, 2016) in Austria and South Africa. Entities that were initially mapped as line or point features were buffered and included with the other polygons when calculating spatial data. Buffer size included in brackets in respective feature class

Habitat classification	Austria			South Africa	
	Landscape entities	Feature class	EUNIS code	Landscape entities	Feature class
Natural/seminatural habitats (SNH)	Fallow	Polygon	I1.5	Fallow	Polygon
	Field and road margins	Line (1 m)	X07	Natural/seminatural (fynbos, renosterveld, or wetlands)	Polygon
	Hedgerow	Polygon	FA	Hedgerow	Polygon
	Sparsely wooded grassland	Polygon	E7	Natural grazing	Polygon
	Grassland	Polygon	E		
	Meadow orchard	Polygon	G1.D		
	Pasture	Polygon	E2.1		
	Loess walls	Polygon	NA		
	Wetland	Polygon	D		
Woody structures	Woodland and forest	Polygon	G	Closed canopy stands of woody vegetation (mostly invasive alien trees)	Polygon
	Tree rows	Line (2 m)	G5.1	Tree rows	Polygon
	Solitary trees	Point (2 m)	NA	Solitary trees (mostly invasive alien trees)	Point (2 m)
Vineyard	Vineyard	Polygon	FB4	Vineyard	Polygon
Agriculture	Annual insect‐pollinated crops	Polygon	I1	Planted pasture	Polygon
	Annual wind‐pollinated crops	Polygon	I1.1	Olive orchard	Polygon
				Deciduous fruit orchard	Polygon
				Vegetables	Polygon
Artificial and constructed entities	Roads, gravel and dirt roads, traffic areas	Polygon	J4.2	Unsealed surfaces (unsealed roads, unvegetated areas)	Polygon
	Settlements	Polygon	J2	Sealed roads	Polygon
	Towns and villages	Polygon	J1	Rail roads	Polygon
	Industrial sites	Polygon	J	Built‐up areas (e.g., buildings)	Polygon
Water body	Ponds and rivers	Polygon	C	Dams, ponds, and rivers	Polygon

For further analysis, landscape features were aggregated to habitat classes (Table 3): SNHs only included landscape features characteristic for open areas (e.g., natural and seminatural grasslands, natural sclerophyllous vegetation, fynbos, fallow land, grass strips, hedges). In Austria, SNHs include a greater proportion of seminatural elements, whereas in South Africa, SNHs represent more pristine habitats such as the fynbos and renosterveld vegetation. Woody structures (e.g., woodlots, tree rows, alien tree stands, forests) were pooled in a separate class for several reasons: Firstly, most bee species colonize open habitats with many of those subsumed in SNH. Secondly, woody structures may be particularly important for aboveground nesting bee species. Thirdly, woody structures represent different characteristics in the two countries. While in Austria, they consist mostly of native tree species and represent seminatural structures (Burgenländische Landesregierung, 2018), in South Africa, a high proportion of introduced, invasive tree species (pine, eucalyptus, and acacia species) are present in the woody structures, with natural woodland uncommon in the shrub‐dominated fynbos vegetation (Melin et al., 2018; Rebelo et al., 2006).

### 2.4 Data analysis

Numerical variables of repeated measurements per study vineyard were aggregated (species numbers of wild bees and flowering plants) or averaged (mean plant species aggregated across seasons, mean vegetation cover). The only exception was one vineyard in Austria, which was permanently vegetated in 2015, but tilled in early spring 2016, and therefore, the two years were treated as separate observations. Honey bees (*Apis mellifera*) were excluded from analysis, because managed hives may lead to biased results. Additionally, brood–parasitic wild bee species were excluded from analysis, because they predominantly depend on the occurrence of the host species and no parasitic bee species were reported in the South African vineyards. Except for data standardization with z‐scores, all statistical analysis was performed in R (R Core Team, 2019).

Functional trait richness and community‐weighted means (CWMs) of wild bees and flowering plants were calculated with the function *dbFD* in the R package *FD* (Villéger et al., 2008; Laliberté et al., 2015). The calculation of wild bee FRic included all functional traits (Table 2). To estimate the flowering plants’ FRic, presence/absence data were used, because the cover of flowering plant species was not assessed in all studied vineyards. The *Cailliez* correction method was applied to avoid biased estimations of FRic of wild bees and flowering plants (Laliberté et al., 2015). As only categorical traits were selected to represent flower traits (Table 2), FRic was measured as the number of unique trait combinations per vineyard. In order to include floral traits as predictor variables, the dominant class (i.e., CWM) per floral trait (Table 2) and vineyard was obtained from the *dbFD* function.

To analyze the effects and possible interaction of environmental filters at different spatial scales on wild bees and flowering plants, the datasets were analyzed together with linear mixed models. To enable the joint statistical analysis of the two countries, and to account for the differences in methodology and sampling effort, numerical variables (*y*) (i.e., count data of wild bee species, flowering and mean plant species richness, FRic indices, inter‐row vegetation cover, proportion of SNHs, and woody structures) were standardized per vineyard (*i*) and country (*j*) by calculating z‐scores (*z_ij_* = (*y_ij_* – *y̅_J_)* /SD*_j_*). The z‐scores do not modify the relationship between response and predictor variables (c.f. Dainese et al., 2019; Garibaldi et al., 2015).

For each response variable (wild bee species richness and FRic, FRic of flowering plants), we fitted a model set of linear mixed‐effect models (i.e., generalized linear models of the Gaussian family) using the R packages *lme4* (Bates et al., 2015) and *nlme* (Pinheiro et al., 2020). One model set either included single predictors (summarized in Table 4) or a priori defined combination and the interaction of two noncollinear predictors (e.g., farm type × SNH) according to our research questions. The flower trait CWMs were also included in the models for wild bees. To account for spatial autocorrelation, we included the locality (i.e., the landscape buffer IDs for AT data; localities with plot pairs for SA data) as random structures in the models. Although using the *z*‐score standardization method, the country effect should be ruled out, we included country as fixed effect in models per set, but none of these models turned out to be most parsimonious. As the flowering plant FRic was strongly correlated with the total species richness of flowering plants (Spearman's correlation: *p* < .01, *r* = .64), we did not model total flowering plant species richness. Model selection was based on the second‐order Akaike information criterion, which corrects for small sample sizes (AICc; Motulsky & Christopoulos, 2003). The cutoff to evaluate the most parsimonious models for a response variable was set at ΔAICc ≤ 2 (R package *AICcmodavg*; Mazerolle, 2016). Model quality of the highest ranked models was assessed by diagnostic plots (R package *DHARMa;* Hartig, 2017) and by calculating the conditional and marginal *R* ^2^ (R package *MuMIn;* Barton, 2016). Additionally, the variance inflation factor (VIF) was calculated for the models with two predictors, to ascertain correct parameter estimation and absence of collinearity between predictors (Zuur et al., 2010). VIFs <3 were considered as absence of collinearity in the models. Graphical visualization of the best model was performed with the *effects* package (Fox, 2018).

**TABLE 4:** Summary of predictors included in mixed models of species richness and functional richness

Spatial scale	Variable	Description
Local	Farm type	Management type. Either organic or conventional.
	Inter‐row vegetation cover	Percentage vegetation cover in vineyard inter‐rows.
	Species richness	Mean amount of flowering plant species per vineyard inter‐row.
	FRic	Functional trait richness (FRic). For response variables related to wild bees, FRic represents functional richness of flowering plants. For response variables related to flowering plants, FRic represents functional richness of wild bees.
	Flower trait CWMs	Community‐weighted means (CWMs) of flowering plants. Only included in models of response variables related to wild bees.
Landscape	SNH	Proportion of natural and seminatural habitat (SNH) in landscape within 500‐m radius of a sample vineyard.
	Woody structures	Proportion of woodlots, tree rows, forests, and solitary trees in landscape within 500‐m radius of a site.

To analyze country‐specific associations of different functional bee traits and flower traits, and their dependence to vineyard management intensity and landscape parameters, a fourth‐corner analysis was conducted. Using the function *traitglm* in the R package *mvabund* (Wang et al., 2012, 2020), we modeled functional bee and flowering plant traits together with vineyard and landscape parameters (not included in flower trait models). Additionally, a LASSO penalty was applied to reduce the environmental–trait relationships to 0 when the correlation was small, which ultimately improves the interpretability of the models’ result. The bee trait models were fitted with negative binomial distribution and the flower trait models with binomial distribution. To study comparable patterns affecting bee and plant traits in the Northern and Southern Hemispheres, each country was analyzed separately with nonstandardized data. Sociality and lecty were excluded from the South African analyses due to missing trait information for many of the morphospecies (mainly from the Halictidae family, which are not fully described in the region, and no species identification keys are available). Flowering seasonality was excluded from the flower trait models (both countries), because some species were only recorded at genus level. For nominal trait variables with two categories (e.g., eusocial and solitary), the results for one category are presented, because the results are the inverse for the two variables.

## 3 RESULTS

Including honey bees and parasitic species, a total of 122 bee species and 3,202 bee individuals (1,390 were honey bees) were documented in vineyards of the two countries: in Austria, 96 spp. and 1,188 individuals (369 honey bees); and in South Africa, 28 spp. and 2014 individuals (1,021 honey bees). The honey bee (*Apis mellifera*) was the only species that occurred in both countries, but was represented by different subspecies. Brood–parasitic bee species (10 species; 21 individuals) were only documented in Austrian vineyards. After excluding the honey bees, *Lasioglossum marginatum* was most abundant (198 specimens) in Austria, followed by *L*. *lineare* (71 specimens) and *Bombus lapidarius* (70 specimens), all three species are eusocial bee species. In South Africa, *Andrena notophila* (611 specimens) and *Tetraloniella junodi* (65 specimens), both solitary bee species, were most abundant after excluding the honey bees. Overall, 94 flowering plant species (Austria: 80; South Africa: 15) were reported and *Plantago lanceolata* was the only flowering plant that occurred in both countries. The most frequent insect‐pollinated flowering plant species in Austria were *Taraxacum officinale* agg., *Convolvulus arvensis*, *Veronica persica*, *Lamium purpureum*, and *Achillea millefolium* flowering in more than 85% of the studied inter‐rows. In South Africa, *Raphanus raphanistrum*, *Senecio* spp., and *Vicia benghalensis* were flowering in 70%–90% of the investigated inter‐rows.

### 3.1 Common drivers of species and functional richness in vineyards across countries

Overall, regression models revealed a strong relationship between FRic of wild bees and insect‐pollinated flowering plants, with these variables improving the model fit considerably. Furthermore, vineyard management and less importantly landscape parameters played an important role in wild bee models. This was not the case for flowering plant FRic, which was only affected by vineyard‐scale parameters.

**FIGURE 3:**
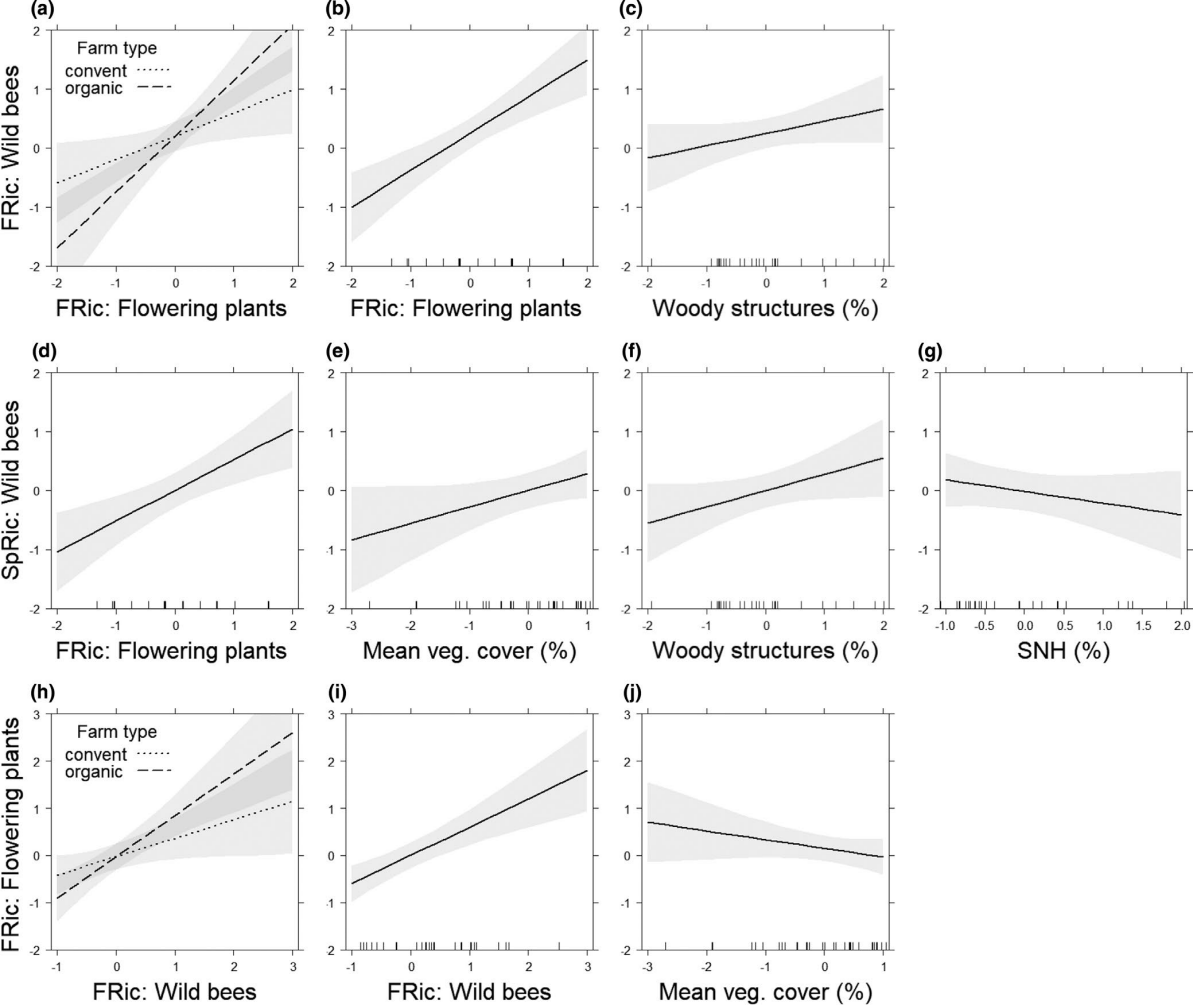
Results for the two countries combined: Wild bee FRic (a–c) in response to the interaction of flowering plant functional richness (FRic) and farm type (organic vs. conventional), flowering plant FRic, and woody structures. Wild bee species richness (SpRic) (d–g) in response to flowering plant functional richness (FRic), inter‐row vegetation cover, proportion of woody structures, and proportion of SNHs at the landscape scale. Flowering plant functional richness (FRic) (h–j) in response to the interaction of farm type and wild bee FRic, wild bee FRic, and inter‐row vegetation cover. All numerical variables were standardized by z‐scores prior to analysis. Gray shading: 95% confidence intervals

The regression analysis revealed three equally parsimonious models explaining wild bee FRic in vineyards (Table 5), highlighting the strong positive effect of increasing flowering plant FRic interacting with organic versus conventional vineyard management (Figure 3a; Table S3). The strong positive relationship between bee and plant FRic is further underlined by the solely positive effect of flowering plant FRic on wild bee FRic (Figure 3b; Table S3). At the landscape scale, the increasing proportion of woody structures had a weak positive effect on wild bee FRic (Figure 3c; Table S3).

**TABLE 5:** Most parsimonious models for wild bee functional richness (FRic), wild bee species richness and flowering plant FRic including respective AICc values, marginal *R* ^2^, and conditional *R* ^2^. Most parsimonious model highlighted in bold. Intercept‐only models: ~1. Random variables are landscape buffer IDs for Austrian models and the localities with plot pairs in South African models

Model	AICc	R^2^m	R^2^c
Wild bee FRic			
**~ flowering plant FRic : farm type**	**86.65**	**0.47**	**0.47**
~ flowering plant FRic	88.41	0.40	0.40
~ flowering plant FRic + woody structures	88.63	0.45	0.45
~ 1	101.76	0.00	0.30
Wild bee species richness			
**~ flowering plant FRic + inter‐row vegetation cover**	**95.75**	**0.31**	**0.43**
~ flowering plant FRic	96.32	0.25	0.51
~ flowering plant FRic + woody structures	96.86	0.30	0.35
~ flowering plant FRic + SNHs	97.65	0.30	**0.52**
~ 1	103.1	0.00	0.21
Flowering plant FRic			
~ **farm type** **: wild bee FRic**	**90.31**	**0.42**	**0.43**
~ wild bee FRic	90.87	0.37	0.37
~ inter‐row vegetation cover + wild bee FRic	91.71	0.40	0.40
~ 1	101.3	0.00	0.30

Abbreviations: AICc, Akaike information criterion corrected; *R^2^m*, marginal *R* ^2^; *R^2^c*, conditional *R* ^2^

Wild bee species richness, for the four models, were equally parsimonious (Table 5). They revealed similar predictors as for wild bee FRic, with positive effects of flowering plant FRic (Figure 3d; Table S3), as well as an increase in vegetation cover in the inter‐rows improving wild bee species richness (Figure 3e; Table S3). At the landscape scale, wild bee species richness was positively affected by increasing proportions of woody structures (Figure 3f; Table S3) and negatively affected, although weakly, by proportion of SNHs (Figure 3g; Table S3).

The flowering plant FRic was equally well explained by three models (Table 5), which did not include any landscape parameter. The results highlight a positive effect of organic vineyard management interacting with increasing wild bee FRic (Figure 3h; Table S3). Wild bee FRic increased flowering plant FRic (Figure 3i; Table S3), whereas increasing inter‐row vegetation cover had a weak negative effect on the FRic of flowering plants (Figure 3j; Table S3).

### 3.2 Unique characteristics of bee and flowering plant functional traits in Austrian and South African vineyards

In both countries, most flowering plant taxa in the vineyard inter‐rows had white or yellow flowers with radial symmetry and belonged to the Asteraceae and Fabaceae family. The nectar of most of the flowering plant species was totally hidden in the flower, and flag, ray, disk, and ray–disk blossoms were the predominant flower shapes. In Austria, the inter‐row vegetation mainly flowered from early summer to mid‐summer, but some vineyards were covered by a high proportion of plant species that potentially flower the whole vegetative period (e.g., *Stellaria media*, *Veronica persica*). In South Africa, inter‐row flowering was predominantly in spring (see Table S4 for CWMs per vineyard).

**FIGURE 4:**
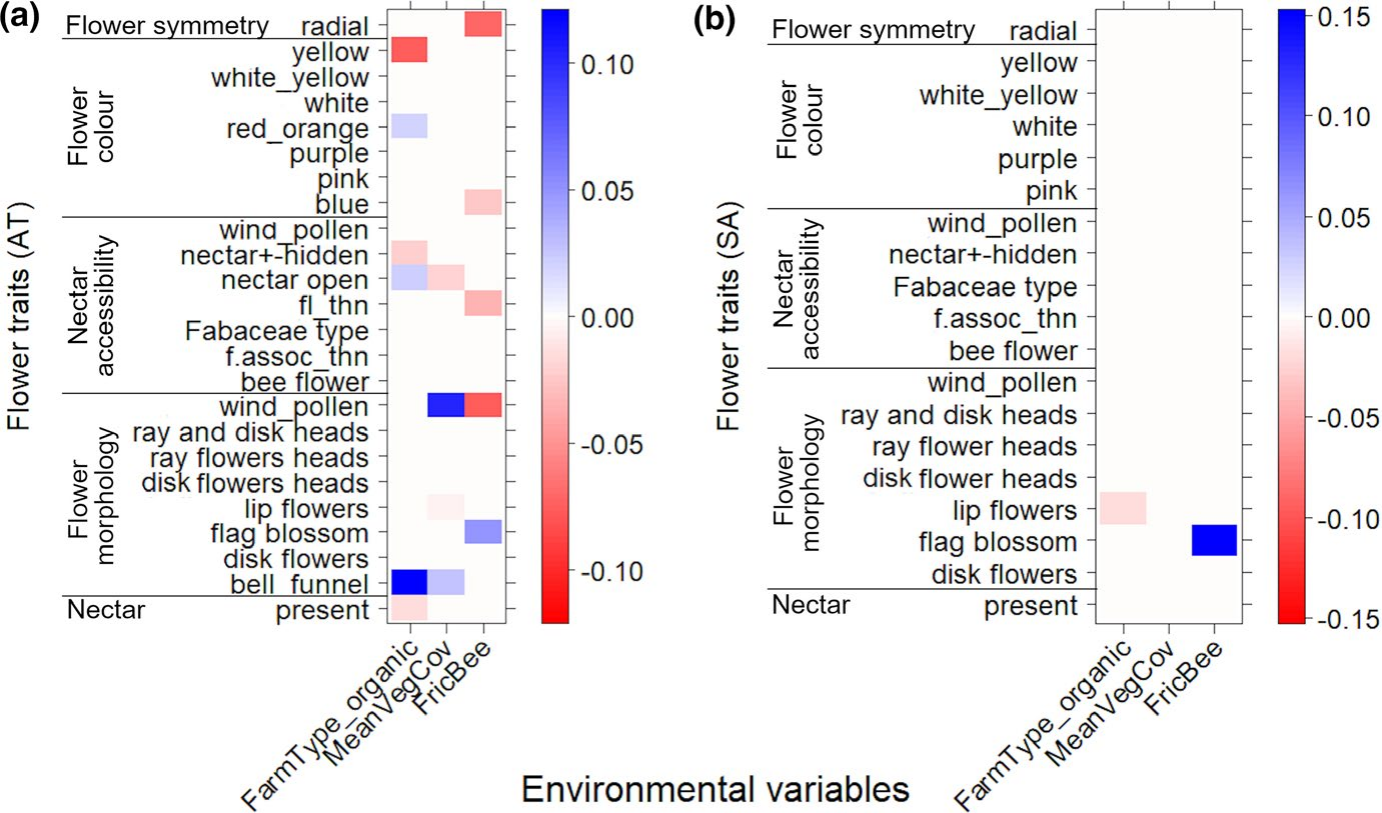
Effects of vineyard management intensity and functional wild bee richness on flower traits in (a) Austrian and (b) South African vineyards. Darker colors represent stronger correlations: red—negative correlation, and blue—positive correlation. Note: If a trait (e.g., flower symmetry) is represented by two categories (e.g., radial and bilateral), only one category is presented in the figure. Abbreviations: fl_thn: flowers with hidden nectar; f.assoc_thn: flower associations with totally hidden nectar; wind_pollen: wind‐pollinated plants and plants with flower providing exclusively pollen; MeanVegCov: mean vegetation cover per study vineyard; FricBee: wild bee functional richness

In organic vineyards in Austria, there was increased abundance of plant species with bell or funnel flowers (coef = 0.12), but decreased abundance of plants with yellow flowers (coef = −0.08) (Figure 4a). Higher inter‐row vegetation cover increased wind‐pollinated plant species or plants where flowers solely provided pollen (coef = 0.1). Plants with radial flowers (coef = −0.07), as well as flowers providing only pollen, and wind‐pollinated plants (coef = −0.08) were negatively associated with increasing wild bee FRic. Fabaceae with flag blossoms were positively associated with high wild bee FRic in Austrian (coef = 0.06) and South African vineyards (coef = 0.15) (Figure 4b).

The bee assemblages in both countries were characterized by a high proportion of ground nesting (Austria: 68%, South Africa: 78%), solitary (Austria: 69%, South Africa: 59%), and polylectic wild bee species (Austria: 85%, South Africa: 100%). However, in Austrian vineyards, eusocial wild bees were more abundant (over 60% of the specimens) than in South Africa (11% of the specimens). In South Africa, the sociality of seven species (59 specimens) and the lecty of 2 species (15 specimens) remain unclear. There were 13 oligolectic species (89 specimens) reported from Austrian inter‐rows, but none were documented here for South African vineyards. Wild bees ITD ranged between 1.58 and 2.61 mm, and the mouthpart length varied between 2.18 and 4.71 mm (see Table S4 for CWMs per vineyard). In comparison with South Africa, the CWM of the Austrian bee assemblages was characterized by smaller (Austria: 2.06 ± 0.31, South Africa: 2.35 ± 0.22) but longer tongued bee species (CWM of proboscis length, mean ± *SD* per study vineyard: Austria: 3.23 ± 0.79, South Africa: 2.99 ± 0.41).

**FIGURE 5:**
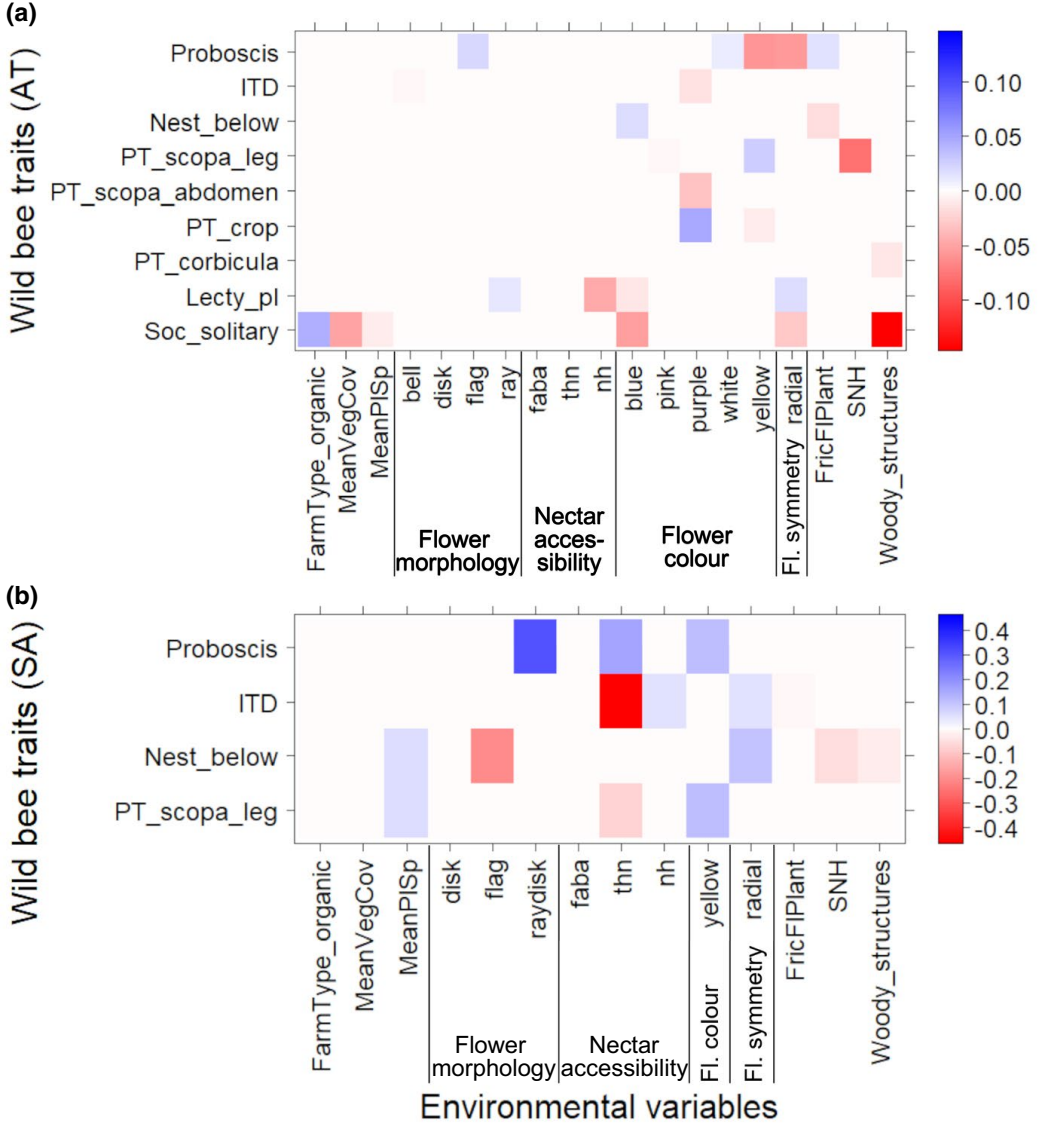
Effects of vineyard management, landscape composition, and flower traits on wild bee traits in (a) Austrian and (b) South African vineyards. Darker colors represent stronger correlations: red—negative correlation; blue—positive correlation. Note: Characteristic flower traits per vineyard based on community‐weighted means; thus, not all trait categories listed in Table 2 are present. If a trait (e.g., bee sociality) is represented by two categories (e.g., eusocial and solitary), only one category is presented in the figure. Abbreviations: ITD: intertegular distance; PT: pollen transport type; pl: polylectic; Nest_below: below nesting species excavate nests into the ground; MeanVegCov: mean vegetation cover per inter‐row; MeanPlSp: mean plant species richness per inter‐row; bell: bell and funnel flowers; disk: disk flowers; flag: flag blossom; ray: Asteraceae, only ray flower heads; h_raydisk: Asteraceae, ray and disk flower heads; faba: Fabaceae type; fl.assoc_thn: flower associations with totally hidden nectar; thn: flowers with totally hidden nectar; nh: nectar ±hidden; FricFlPlant: flowering plant functional richness, SNH: seminatural habitat

The fourth‐corner analysis of Austrian vineyards (Figure 5a) showed low correlation between the analyzed wild bee traits and the environmental variables. An increasing frequency of blue flowers in the inter‐rows correlated with a lower abundance of solitary bee species (coef = −0.06). Purple flowers decreased abundance of bees collecting pollen on the abdomen (coef = −0.04), but benefitted crop‐collecting species (coef = 0.05). Yellow flowers (coef = −0.06) and flowers with radial symmetry (coef = −0.06) were associated with lower abundances of long‐tongued wild bee species (thus increased short‐tongued species). Vineyard management and the proportion of woody structures were associated with a higher abundance of bee species with different social organizations. Solitary species were likely more abundant in organic (coef = 0.05) than in conventional vineyards, but tended to decrease with increased inter‐row vegetation cover (coef = −0.05) and woody structures (coef = −0.15) in the surrounding landscape.

The wild bees in South African vineyards showed different associations with flower traits, management intensity, and landscape composition compared with the Austrian vineyards (Figure 5b). Long‐tongued bees were more abundant in vineyards where plant with ray–disk flower heads (e.g., Asteraceae; coef = 0.3), plants with flower associations with totally hidden nectar (coef = 0.16), and yellow flowers (coef = 0.11) were present. In contrast, ITD decreased with the presence of plants with flower associations with totally hidden nectar (coef = −0.46). At the landscape scale, a higher proportion of SNHs (coef = −0.06) and woody structures (coef = −0.03) slightly decreased belowground nesting species. These species showed a positive association with plants with radial flower symmetry (coef = 0.12) and an increased mean plant species richness (coef = 0.06). The abundance of bee species collecting pollen on a scopa on legs increased with a higher mean plant species richness (coef = 0.05) but was negatively correlated with plants with completely hidden nectar (coef = −0.08).

## 4 DISCUSSION

Functional richness of wild bees and insect‐pollinated flowering plants showed strong positive associations in vineyards. Organic vineyard management increased the positive response to flowering plant FRic. As different pollinators are associated with distinct floral traits (Blüthgen & Klein, 2011), functionally diverse plant communities will likely increase pollinator diversity through complementarity and through augmenting floral resources over time (Balzan et al., 2014). Here, flowering plant FRic enhanced wild bee pollinator species richness and FRic across regions and management regimes. This was supported by results from the fourth‐corner analyses that showed varied associations of bee traits with flowering plant traits.

### 4.1 Common drivers of bee diversity in vineyards across countries

The interacting effect of plant FRic and organic farming on bee FRic suggests that maintaining diverse plant communities in inter‐rows may also enhance other biodiversity‐friendly practices. Compared with organic vineyards, conventional vineyards had higher bee FRic at low levels of flowering plant FRic. This indicates bees may be able to exploit resources better at low levels of flowering plant FRic, likely due to trait matching. For example, we showed that in Austria, yellow flowers are more frequent in conventional vineyards and are beneficial for short‐tongued bees. In South Africa, yellow flowers promoted bees with a tibial scopa and long‐tongued bees, which contributes to increased wild bee FRic. However, a direct positive effect on yellow flowers by conventional vineyard management in South Africa was not detected.

Functional richness and species richness are orthogonal to each other, FRic either increases or remains the same with increasing species richness (Mason et al., 2005; Schleuter et al., 2010). In this study, FRic increased with higher species richness, which explains the similar results of wild bee species richness and FRic of wild bees. The positive effect of higher vegetation cover on bee species richness was also reported for wild bees in other crop systems (Nicholson et al., 2017; Shuler et al., 2005) and vineyards (Kratschmer et al., 2019), as well as for other beneficial organisms (Buchholz et al., 2017; Fiera et al., 2020; Hall et al., 2020; Winter et al., 2018). Benefit is derived from undisturbed soil conditions for eusocial species (Williams et al., 2010), which are predominantly ground nesting species in our study. Additionally, eusocial bee reproduction often depends on a single fertile female for the whole colony, which makes them more vulnerable to soil disturbance (Kratschmer et al., 2018). The positive effect of undisturbed soil conditions on wild bee FRic was not detected in models that combined both countries. Possibly because the 23 ground nesting and eusocial species belong to the Halictidae family (*Halictus* spp. and *Lasioglossum* spp.) and their contribution to FRic is low, as most are very small, short‐tongued species that collect pollen with a tibial scopa.

In the current study, we did not see evidence that higher proportions of SNHs compensate for the negative effects of intensive vineyard management. However, higher proportions of woody structures at the landscape scale increased wild bee FRic in the study vineyards in both countries, but this has to be interpreted with care, because the characteristics of the woody structures vary across the study regions. In Austria, large woody structures represent seminatural structures of species‐rich thermophilic oak and oak‐hornbeam forests partly belonging to the European Natura 2000 protected area network (Burgenländische Landesregierung, 2018). Although only one third of the Austrian bee species found in vineyards were eusocial, this group contributed to functional richness, with the species varying in nesting type, body size, mouthpart length, and pollen collection with different structures (e.g., *Bombus* spp vs. *Lasioglossum* spp.). In the Cape Floristic Region, natural habitats (SNHs) consist of low‐growing shrubs with few trees. The woody structures recorded here consisted mainly of non‐native invasive species, which probably do not provide habitat for many of the native bee species, as plant species richness and ground‐dwelling arthropod diversity decline under stands of alien trees (Magoba & Samways, 2012; Richardson et al., 1989; Schoeman & Samways, 2011). However, dead‐wood nesting species, such as *Xylocopa rufitarsis*, *Lithurgus spiniferus,* or *Allodape tridentipes*, may find suitable nesting sites in these wooded areas. Additionally, certain flowering alien trees, such as *Acacia saligna* and *Eucalyptus cladocalyx*, can provide floral resources to local pollinators (Gibson et al., 2013; De Lange et al., 2013).

The models revealed a weak negative effect of SNHs on wild bee species richness. However, this result should be interpreted with caution (Kratschmer et al., 2018), as either a pull effect due to good habitat quality or, in contrast, a generally poor habitat quality of the SNHs could be responsible for this result. As Kehinde and Samways (2014a) found higher bee and flowering plant diversity in natural fynbos sites compared with vineyards, the pull‐effect explanation is probably more likely in South Africa. It also explains why only 28 bee species were documented in vineyards in a country known as a bee diversity hot spot. We cannot underpin the pull‐effect explanation directly for Austria, but the importance of SNHs over vineyards as habitat for wild bees was recently reported (Pascher et al., 2020). Another reason for the low species diversity could lie in the different sampling methods and frequencies in the two countries. Although the combination of sampling methods used in South Africa (trapping, transect sampling) should provide a representative sample of the bee community (Prendergast et al., 2020; Schindler et al., 2013; Vrdoljak & Samways, 2012), the very short activity period of many bee species in combination with the short sampling period may have missed many bees in the South African vineyards. Also, different sampling methodology used in the two countries may have influenced the traits recorded, especially those selected from the colored pan traps (McCravy et al., 2019).

### 4.2 Common drivers of flowering plant diversity in vineyards between countries

Functional richness of flowering plants increased with wild bee FRic, organic management, and lower vegetation cover. The positive effect of higher bee species richness on insect‐pollinated plant species richness was also shown by Papanikolaou et al. (2017) across 24 European study sites. Furthermore, they also did not detect any effect of landscape composition on functional richness of insect‐pollinated plants. It seems contradictory that less intensive management associated with higher vegetation cover results in lower insect‐pollinated plant FRIc. Most likely the effect is related to the higher proportion of grasses dominating vineyard inter‐rows with permanent vegetation cover, as reported from vineyards across Europe (Hall et al., 2020). In accordance with the intermediate disturbance hypothesis (Connell, 1978), infrequent and alternating tillage in vineyard inter‐rows increases species richness. Just like wild flower strips, which tend to be dominated by grasses without disturbance (Schmid‐Egger & Witt, 2014), vineyard inter‐rows should also be occasionally tilled to increase cover by ruderal plants (Hall et al., 2020) and less competitive species (Gago et al., 2007), which contribute to the resource provision by a diverse flowering plant community.

The negative effect of conventional management on vascular plant species richness due to the use of herbicides in vineyards was shown for Italian vineyards (Nascimbene et al., 2012) and in an experimental field trial in California (Sanguankeo & León, 2011). Without the use of herbicides in conventional vineyards, Bruggisser et al. (2010) and Kehinde and Samways (2014a) did not find any significant difference between management type on plant species richness. It is surprising that in this study, where winegrowers did not use any herbicides in conventional vineyard inter‐rows, organic viticulture still increased FRIc of flowering plants.

Despite the fact that *Plantago lanceolata* was the only flowering plant species documented in inter‐rows in both countries, several taxa occur in both countries. This larger overlap is due to the high proportion of alien species (43% of all flowering plants) found in South African vineyards: *Echium plantagineum, Medicago polymorpha, Helminthotheca echioides, Raphanus raphanistrum, Trifolium angustifolium,* and *Vicia benghalensis*. *Raphanus raphanistrum* and *V. benghalensis* were probably introduced through seed mixtures.

### 4.3 Unique characteristics of bee and flowering plant functional traits in Austrian and South African vineyards

Apart from the previously discussed effects of flower traits on wild bee functional traits, the detailed trait analysis provided additional and country‐specific patterns, which can be explained by trait matching. However, due to the low correlation between traits and environmental variables (especially in Austria) these results should be interpreted with care. It is important to keep in mind that only plant occurrence data, but not flower abundance or cover, were available for this analysis. Thus, clearer trait matching patterns may be derived from plant–pollinator interaction data; however, those data were not available. Further, it should be noted that bees flower color perception is shifted toward the UV light spectrum. In this study, flower colors, as perceived by humans, were used, due to missing information about flower UV reflectance for >25% of the plant species considered in our study. As pointed out by Burr and Barthlott (1993) and Burr et al. (1995), there exists a relationship between these different color perceptions, which supports the selection of flower colors as perceived by humans used for this analysis.

In Austria, yellow flowers and radial flower symmetry, which are predominantly plants belonging to the Asteraceae family, benefitted short‐tongued bee taxa. The nectar of Asteraceae flowers is not easily available, but hidden in each flower, which excludes other, less reliable pollinators such as flies from collecting it. Short‐tongued bees (e.g., *Andrena*, *Lasioglossum,* or *Halictus* species, Figure S2) are matched with these flower traits and able to collect nectar and pollen from the flowers efficiently (Mani & Saravanan, 1999). In contrast to the Austrian results, long‐tongued bee species benefitted from Asteraceae flowers in South African vineyards. Although no flower abundance data are available, we cannot rule out that yellow Asteraceae predominantly flowered in the South African inter‐rows and that long‐tongued bee species (e.g., *Xylocopa rufitarsis*, *Tetraloniella junodi*) collect mainly pollen from these flowers (Mani & Saravanan, 1999). However, it is puzzling that although Fabaceae species flowered in most of the South African inter‐rows, tongue length was not associated with the specific flower morphology or nectar accessibility, especially as these plants would seem to be ideal for long‐tongued bee species. We propose the following explanations: Firstly, the reported bee species with longer mouthparts (> 4 mm) may not prefer or be adapted to collecting floral resources from the introduced Fabaceae plants. Secondly, the 2010 dataset also includes plant–pollinator interactions (Kehinde & Samways, 2014c), which reveals that Fabaceae species were most frequently visited by *Apis mellifera*. This could indicate either a niche shift of other bee species to other plant species due to competition with the honey bees, or that honey bees are more adaptable to using introduced plant species compared with the other wild bee species (Mallinger et al., 2017).

## 5 CONCLUSION

We found strong relations between the functional richness (FRIc) of insect‐pollinated plants and wild bees in Austrian and South African vineyards. Therefore, in order to promote wild bee functional richness and diversity it would be essential to increase flowering plant FRic in vineyards by adapting vineyard management practices, such as the selection of cover crop mixtures and implementation of infrequent tillage that favor a wide variety of plant traits. Cover crop selection with diverse mixtures of floral traits including native species to increase biodiversity and plant–pollinator networks in vineyards might also be considered in agrienvironmental programs and certification requirements for wine marketing. Furthermore, the establishment and maintenance of habitats such as woody structures, encouraging native tree and shrub species at the landscape scale should also be considered in order to maintain wild bee diversity. Our findings provide important information that agroecological vineyard management in both regions can promote general wild bee diversity even though grapevines are not dependent on insect pollination.

## CONFLICT OF INTEREST

The authors declare no competing financial interests.

## AUTHOR CONTRIBUTIONS

**Sophie Kratschmer:** Data curation (lead); Formal analysis (lead); Investigation (lead); Methodology (equal); Supervision (lead); Writing‐original draft (lead); Writing‐review & editing (equal). **Bärbel Pachinger:** Conceptualization (equal); Investigation (equal); Methodology (equal); Writing‐review & editing (equal). **René Gaigher:** Data curation (lead); Formal analysis (equal); Project administration (equal); Supervision (equal); Writing‐original draft (equal). **James S. Pryke:** Methodology (equal); Supervision (equal); Writing‐review & editing (equal). **Julia van Schalkwyk:** Data curation (lead); Methodology (equal); Supervision (equal); Validation (equal); Writing‐review & editing (equal). **Michael J. Samways:** Conceptualization (equal); Investigation (equal); Methodology (equal); Supervision (equal); Writing‐review & editing (equal). **Annalie Melin:** Methodology (equal); Resources (equal); Validation (equal); Writing‐review & editing (equal). **Temitope Kehinde:** Data curation (equal); Formal analysis (equal); Investigation (lead); Writing‐review & editing (equal). **Johann G. Zaller:** Funding acquisition (equal); Resources (equal); Writing‐review & editing (equal). **Silvia Winter:** Conceptualization (equal); Formal analysis (equal); Investigation (equal); Methodology (lead); Project administration (lead); Supervision (equal); Writing‐original draft (equal).

## Supporting information

Supplementary Material

## Data Availability

Data are available via Zenodo: https://doi.org/10.5281/zenodo.4686869.
