# Supplementary material for: Enhancing flowering plant functional richness improves wild bee diversity in vineyard inter‐rows in different floral kingdoms
Source: Ecol Evol. 2021 May 4;11(12):7927–45. doi: 10.1002/ece3.7623 (PMC8216979; doi:10.1002/ece3.7623)
Supplement: Supplementary file 1 — Supplementary Material [file ECE3-11-7927-s001.docx]

**Appendix - Tables**

Table S1: Wild bee species with total abundance per species, including functional traits, mean ITD and mouthpart (Proboscis) lengths calculated with the R package “bee IT” (Cariveau *et al.*, 2016; Melin *et al.*, 2019) and revised with the R package *pollimetry* (Kendall, 2018).

| **Species** | **Family** | **Country** | **Nesting** | **Lecty** | **Sociality** | **ITD (mm)** | **Pollen transport** | **Proboscis (mm)** | **Seasonality** | **Total abundance** |
| --- | --- | --- | --- | --- | --- | --- | --- | --- | --- | --- |
| **Austria & South Africa** | | | | | | | | | | |
| *Apis mellifera* LINNAEUS 1759 | Apidae | AT & RSA | Above | pl | social | 2.68 | Corbicula | 5.51 | whole veg. Period | 1390 |
| **Austria** | | | | | | | | | | |
| *Andrena blüthgeni* Stöckert 1930 | Andrenidae | AT | Below | pl | solitary | 2.80 | Scopa_leg | 2.87 | whole veg. Period | 1 |
| *Andrena danuvia* Stöckert 1950 | Andrenidae | AT | Below | pl | solitary | 2.46 | Scopa_leg | 2.52 | spring | 1 |
| *Andrena dorsata* (KIRBY 1802) | Andrenidae | AT | Below | pl | solitary | 2.04 | Scopa_leg | 2.11 | spring_summer | 2 |
| *Andrena flavipes* PANZER 1800 | Andrenidae | AT | Below | pl | solitary | 2.12 | Scopa_leg | 2.19 | spring_summer | 19 |
| *Andrena fulvago* (CHRIST 1791) | Andrenidae | AT | Below | ol | solitary | 2.15 | Scopa_leg | 2.22 | summer | 1 |
| *Andrena gravida* IMHOFF 1833 | Andrenidae | AT | Below | pl | solitary | 2.82 | Scopa_leg | 2.89 | spring | 9 |
| *Andrena haemorrhoa* (FABRICIUS 1781) | Andrenidae | AT | Below | pl | solitary | 2.56 | Scopa_leg | 2.63 | spring_summer | 1 |
| *Andrena labialis* (KIRBY 1802) | Andrenidae | AT | Below | ol | solitary | 2.32 | Scopa_leg | 2.39 | summer | 4 |
| *Andrena minutula* (KIRBY) 1803 | Andrenidae | AT | Below | pl | solitary | 2.21 | Scopa_leg | 2.28 | spring_summer | 2 |
| *Andrena nitida* (MÜLLER 1776) | Andrenidae | AT | Below | pl | solitary | 3.04 | Scopa_leg | 3.10 | spring | 2 |
| *Andrena ovatula* (KIRBY 1802) | Andrenidae | AT | Below | pl | solitary | 2.07 | Scopa_leg | 2.14 | spring_summer | 37 |
| *Andrena polita* SMITH 1847 | Andrenidae | AT | Below | ol | solitary | 2.26 | Scopa_leg | 2.33 | summer_late summer | 1 |
| *Andrena simontornyella* NOSKIEWICZ 1940 | Andrenidae | AT | Below | pl | solitary | 1.28 | Scopa_leg | 1.35 | spring_summer | 8 |
| *Anthidium strigatum* (PANZER 1805) | Megachilidae | AT | Above | pl | solitary | 1.01 | Scopa_abdomen | 1.88 | summer | 1 |
| *Anthophora plumipes* (PALLAS 1772) | Apidae | AT | Below | pl | solitary | 3.76 | Scopa_leg | 7.61 | spring | 3 |
| *Bombus haematurus* KRIECHBAUMER 1870 | Apidae | AT | Above | pl | eusocial | 3.65 | Corbicula | 7.40 | whole veg. Period | 1 |
| *Bombus sylvarum* (LINNAEUS 1761) | Apidae | AT | Above | pl | eusocial | 3.03 | Corbicula | 6.19 | whole veg. Period | 4 |
| *Bombus hortorum* (LINNAEUS 1762 | Apidae | AT | Above | pl | eusocial | 3.41 | Corbicula | 6.94 | whole veg. Period | 3 |
| *Bombus lapidarius* (LINNAEUS 1758) | Apidae | AT | Above | pl | eusocial | 4.02 | Corbicula | 8.12 | whole veg. Period | 70 |
| *Bombus pascuorum* (SCOPOLI 1763) | Apidae | AT | Above | pl | eusocial | 3.24 | Corbicula | 6.61 | whole veg. Period | 17 |
| *Bombus terrestris / B. lucorum* | Apidae | AT | Above | pl | eusocial | 3.66 | Corbicula | 7.42 | whole veg. Period | 40 |
| *Ceratina chalybea* CHEVRIER 1873 | Apidae | AT | Above | pl | solitary | 1.83 | Crop | 3.82 | whole veg. Period | 2 |
| *Ceratina cucurbitina* (ROSSI 1792) | Apidae | AT | Above | pl | solitary | 1.20 | Crop | 2.53 | summer | 1 |
| *Ceratina cyanea* (KIRBY 1802) | Apidae | AT | Above | pl | solitary | 1.27 | Crop | 2.68 | spring_summer | 3 |
| *Ceratina nigrolabiata* FRIESE 1896 | Apidae | AT | Above | pl | solitary | 1.33 | Crop | 2.80 | summer_late summer | 4 |
| *Dasypoda hirtipes* (FABRICIUS 1793) | Apidae | AT | Below | ol | solitary | 3.17 | Scopa_leg | 6.47 | summer | 1 |
| *Eucera chrysopyga* (Pérez. 1879) | Apidae | AT | Below | pl | solitary | 3.31 | Scopa_leg | 6.75 | spring_summer | 2 |
| *Eucera longicornis* (LINNAEUS 1758) | Apidae | AT | Below | ol | solitary | 3.21 | Scopa_leg | 6.54 | spring_summer | 1 |
| *Eucera nigrescens* PÉREZ 1880 | Apidae | AT | Below | ol | solitary | 3.17 | Scopa_leg | 6.47 | spring_summer | 15 |
| *Halictus kessleri* BRAMSON 1880 | Halictidae | AT | Below | pl | eusocial | 1.27 | Scopa_leg | 1.72 | whole veg. Period | 8 |
| *Halictus maculatus* SMITH 1849 | Halictidae | AT | Below | pl | eusocial | 1.32 | Scopa_leg | 1.79 | whole veg. Period | 15 |
| *Halictus quadricinctus* (FABRICIUS 1776) | Halictidae | AT | Below | pl | solitary | 2.67 | Scopa_leg | 3.53 | spring_summer | 5 |
| *Halictus rubicundus* (CHRIST 1791) | Halictidae | AT | Below | pl | eusocial | 2.05 | Scopa_leg | 2.73 | whole veg. Period | 1 |
| *Halictus seladonius* (FABRICIUS 1794) | Halictidae | AT | Below | pl | eusocial | 1.48 | Scopa_leg | 2.00 | whole veg. Period | 2 |
| *Halictus simplex/eurygnathus* | Halictidae | AT | Below | pl | solitary | 1.71 | Scopa_leg | 2.29 | whole veg. Period | 32 |
| *Halictus smaragdulus* VACHAL 1895 | Halictidae | AT | Below | pl | eusocial | 1.19 | Scopa_leg | 1.61 | whole veg. Period | 2 |
| *Halictus subauratus* (ROSSI 1792) | Halictidae | AT | Below | pl | eusocial | 1.41 | Scopa_leg | 1.90 | whole veg. Period | 15 |
| *Halictus tumulorum* (LINNAEUS 1758) | Halictidae | AT | Below | pl | eusocial | 1.31 | Scopa_leg | 1.78 | whole veg. Period | 2 |
| *Heriades truncorum* (LINNAEUS 1758) | Megachilidae | AT | Above | ol | solitary | 1.32 | Scopa_abdomen | 2.43 | summer_late summer | 3 |
| *Hylaeus annularis* (KIRBY 1802) | Colletidae | AT | Above | pl | solitary | 1.34 | Crop | 1.14 | summer_late summer | 2 |
| *Hylaeus brevicornis* NYLANDER 1852 | Colletidae | AT | Above | pl | solitary | 1.01 | Crop | 0.87 | summer_late summer | 2 |
| *Hylaeus communis* NYLANDER 1852 | Colletidae | AT | Above | pl | solitary | 0.96 | Crop | 0.83 | summer_late summer | 1 |
| *Hylaeus confusus* NYLANDER 1852 | Colletidae | AT | Above | pl | solitary | 1.17 | Crop | 1.00 | summer_late summer | 1 |
| *Hylaeus gibbus* SAUNDERS 1850 | Colletidae | AT | Above | pl | solitary | 1.26 | Crop | 1.08 | summer_late summer | 3 |
| *Hylaeus gredleri* FÖRSTER 1871 | Colletidae | AT | Above | pl | solitary | 0.90 | Crop | 0.78 | summer | 1 |
| *Hylaeus imparilis* FÖRSTER 1871 | Colletidae | AT | Above | pl | solitary | 0.99 | Crop | 0.85 | summer | 1 |
| *Hylaeus trinotatus* PÉREZ 1895 | Colletidae | AT | Above | pl | solitary | 1.48 | Crop | 1.25 | spring_summer | 1 |
| *Lasioglossum aeratum* (KIRBY 1802) | Halictidae | AT | Below | pl | eusocial | 0.92 | Scopa_leg | 1.26 | whole veg. Period | 3 |
| *Lasioglossum calceatum* (SCOPOLI 1763) | Halictidae | AT | Below | pl | eusocial | 1.74 | Scopa_leg | 2.34 | whole veg. Period | 14 |
| *Lasioglossum discum* (SMITH 1853) | Halictidae | AT | Below | pl | solitary | 2.14 | Scopa_leg | 2.85 | summer | 4 |
| *Lasioglossum glabriusculum* (MORAWITZ 1872) | Halictidae | AT | Below | pl | eusocial | 0.78 | Scopa_leg | 1.08 | whole veg. Period | 7 |
| *Lasioglossum griseolum* (MORAWITZ 1872) | Halictidae | AT | Below | pl | solitary | 1.07 | Scopa_leg | 1.45 | whole veg. Period | 3 |
| *Lasioglossum interruptum* (PANZER 1798) | Halictidae | AT | Below | pl | eusocial | 1.40 | Scopa_leg | 1.89 | whole veg. Period | 1 |
| *Lasioglossum laevigatum* (Kirby 1802) | Halictidae | AT | Below | pl | solitary | 2.21 | Scopa_leg | 2.94 | spring_summer | 1 |
| *Lasioglossum laterale* (Brullé. 1832) | Halictidae | AT | Below | pl | solitary | 1.86 | Scopa_leg | 2.49 | spring | 1 |
| *Lasioglossum laticeps* (Schenck 1869) | Halictidae | AT | Below | pl | eusocial | 1.59 | Scopa_leg | 2.13 | whole veg. Period | 3 |
| *Lasioglossum lativentre* (SCHENCK 1853) | Halictidae | AT | Below | pl | solitary | 1.50 | Scopa_leg | 2.02 | summer_late summer | 6 |
| *Lasioglossum leucozonium* (SCHRANK 1781) | Halictidae | AT | Below | pl | solitary | 1.63 | Scopa_leg | 2.20 | summer_late summer | 3 |
| *Lasioglossum lineare* (SCHENCK 1869) | Halictidae | AT | Below | pl | eusocial | 1.23 | Scopa_leg | 1.67 | whole veg. Period | 71 |
| *Lasioglossum malachurum* (KIRBY 1802) | Halictidae | AT | Below | pl | eusocial | 1.48 | Scopa_leg | 2.00 | whole veg. Period | 13 |
| *Lasioglossum marginatum* (BRULLÉ 1832) | Halictidae | AT | Below | pl | eusocial | 1.78 | Scopa_leg | 2.39 | spring | 198 |
| *Lasioglossum mesosclerum* (PÉREZ 1903) | Halictidae | AT | Below | pl | unknown | 1.22 | Scopa_leg | 1.65 | whole veg. Period | 2 |
| *Lasioglossum minutissimum* (KIRBY 1802) | Halictidae | AT | Below | pl | solitary | 0.80 | Scopa_leg | 1.10 | whole veg. Period | 7 |
| *Lasioglossum nigripes* (LEPELETIER 1841) | Halictidae | AT | Below | pl | eusocial | 1.94 | Scopa_leg | 2.59 | whole veg. Period | 3 |
| *Lasioglossum pauxillum* (SCHENCK 1853) | Halictidae | AT | Below | pl | eusocial | 1.16 | Scopa_leg | 1.58 | whole veg. Period | 6 |
| *Lasioglossum politum* (SCHENCK 1853) | Halictidae | AT | Below | pl | eusocial | 0.91 | Scopa_leg | 1.26 | whole veg. Period | 3 |
| *Lasioglossum punctatissimum* (SCHENCK 1853) | Halictidae | AT | Below | pl | solitary | 1.14 | Scopa_leg | 1.56 | whole veg. Period | 2 |
| *Lasioglossum puncticolle* (MORAWITZ 1872) | Halictidae | AT | Below | pl | eusocial | 1.53 | Scopa_leg | 2.06 | whole veg. Period | 1 |
| *Lasioglossum pygmaeum* (SCHENCK 1853) | Halictidae | AT | Below | pl | solitary | 1.00 | Scopa_leg | 1.36 | whole veg. Period | 4 |
| *Lasioglossum quadrinotatum* | Halictidae | AT | Below | pl | solitary | 1.72 | Scopa_leg | 2.30 | whole veg. Period | 1 |
| *Lasioglossum villosulum* (KIRBY 1802) | Halictidae | AT | Below | pl | solitary | 1.16 | Scopa_leg | 1.58 | whole veg. Period | 2 |
| *Lasioglossum xanthopus* (KIRBY 1802) | Halictidae | AT | Below | pl | solitary | 2.21 | Scopa_leg | 2.94 | spring_summer | 5 |
| *Lasioglossum zonulum* (SMITH 1848) | Halictidae | AT | Below | pl | solitary | 2.18 | Scopa_leg | 2.90 | whole veg. Period | 1 |
| *Megachile circumcincta* (KIRBY 1802) | Megachilidae | AT | Above | pl | solitary | 2.79 | Scopa_abdomen | 5.01 | summer | 1 |
| *Melitta leporina* (PANZER 1799) | Mellitidae | AT | Below | ol | solitary | 1.95 | Scopa_leg | 2.09 | summer | 2 |
| *Osmia aurulenta* (PANZER 1799) | Megachilidae | AT | Above | pl | solitary | 2.89 | Scopa_abdomen | 5.18 | spring_summer | 2 |
| *Osmia caerulescens* (LINNAEUS 1758) | Megachilidae | AT | Above | pl | solitary | 2.17 | Scopa_abdomen | 3.94 | whole veg. Period | 5 |
| *Osmia claviventris* THOMSON 1873 | Megachilidae | AT | Above | pl | solitary | 2.01 | Scopa_abdomen | 3.65 | summer | 2 |
| *Osmia leucomelana* (KIRBY 1802) | Megachilidae | AT | Above | pl | solitary | 1.41 | Scopa_abdomen | 2.59 | summer | 5 |
| *Osmia rufohirta* LATREILLE 1812 | Megachilidae | AT | Above | pl | solitary | 2.12 | Scopa_abdomen | 3.84 | summer | 2 |
| *Osmia spinulosa* (KIRBY 1802) | Megachilidae | AT | Above | ol | solitary | 1.84 | Scopa_abdomen | 3.35 | summer | 2 |
| *Panurgus calcaratus* (SCOPOLI 1763) | Andrenidae | AT | Below | ol | solitary | 1.64 | Scopa_leg | 1.71 | summer | 8 |
| *Rhophitoides canus* (EVERSMANN 1852) | Halictidae | AT | Below | ol | solitary | 1.67 | Scopa_leg | 2.25 | summer | 2 |
| *Systropha curvicornis* (SCOPOLI 1770) | Halictidae | AT | Below | ol | solitary | 1.98 | Scopa_abdomen | 2.64 | summer | 41 |
| *Systropha planidens* GIRAUD 1862 | Halictidae | AT | Below | ol | solitary | 2.29 | Scopa_abdomen | 3.04 | summer | 8 |
| **South Africa** | | | | | | | | | | |
| **Allodape tridentipes* COCKERELL 1933 | Apidae | RSA | Above |  | solitary | 2.17 | Scopa_leg | 4.48 | spring_summer | 14 |
| *Amegilla spilostoma* BROOKS 1988 | Apidae | RSA | Below | pl | solitary | 2.59 | Scopa_leg | 5.33 | spring_summer | 1 |
| *Andrena notophila* COCKERELL 1933 | Andrenidae | RSA | Below | pl | solitary | 2.62 | Scopa_leg | 2.69 | whole veg. Period | 611 |
| **Anthophora diversipes* FRIESE 1922 | Apidae | RSA | Below | pl | solitary | 3.52 | Scopa_leg | 7.15 | spring | 6 |
| **Anthophora wartmanni* FRIESE 1905 | Apidae | RSA | Below | pl | solitary | 3.74 | Scopa_leg | 7.58 | spring | 16 |
| Halictidae sp_10 | Halictidae | RSA | Below | pl | unknown |  | Scopa_leg |  |  | 1 |
| *Halictus* sp. 1_09 | Halictidae | RSA | Below | pl | solitary | 1.00 | Scopa_leg | 1.37 |  | 25 |
| *Halictus* sp.1_10 | Halictidae | RSA | Below | pl | unknown | 1.78 | Scopa_leg | 2.38 |  | 22 |
| *Halictus* sp.2_10 | Halictidae | RSA | Below | pl | unknown | 1.17 | Scopa_leg | 1.59 |  | 17 |
| *Lasioglossum* sp. 1_09 | Halictidae | RSA | Below | pl | eusocial | 1.30 | Scopa_leg | 1.76 |  | 12 |
| *Lasioglossum* sp. 2_09 | Halictidae | RSA | Below | pl | eusocial | 1.38 | Scopa_leg | 1.87 |  | 47 |
| *Lasioglossum* sp. 3_09 | Halictidae | RSA | Below | pl | eusocial | 1.75 | Scopa_leg | 2.35 |  | 2 |
| *Lasioglossum* sp. 4_09 | Halictidae | RSA | Below | pl | eusocial | 2.00 | Scopa_leg | 2.67 |  | 47 |
| *Lasioglossum* sp.1_10 | Halictidae | RSA | Below | pl | unknown | 1.97 | Scopa_leg | 2.63 |  | 1 |
| *Lasioglossum* sp.2_10 | Halictidae | RSA | Below | pl | unknown | 1.08 | Scopa_leg | 1.48 |  | 9 |
| *Lasioglossum* sp.3_10 | Halictidae | RSA | Below | pl | unknown | 1.73 | Scopa_leg | 2.32 |  | 5 |
| *Lasioglossum* sp.4_10 | Halictidae | RSA | Below | pl | unknown | 2.05 | Scopa_leg | 2.73 |  | 4 |
| *Lithurgus spiniferus* CAMERON 1905 | Megachilidae | RSA | Above | pl | solitary | 2.66 | Scopa_abdomen | 4.78 | spring_summer | 8 |
| *Megachile harthulura* COCKERELL 1937 | Megachilidae | RSA | Above | pl | solitary | 2.36 | Scopa_abdomen | 4.26 | whole veg. Period | 1 |
| *Melitta arrogans* (SMITH 1916) | Melittidae | RSA | Below | pl | solitary | 3.47 | Scopa_leg | 3.63 | whole veg. Period | 1 |
| *Patellapis* sp. 1_09 | Halictidae | RSA | Below | pl | solitary | 1.65 | Scopa_leg | 2.22 |  | 60 |
| *Patellapis* sp.1_10 | Halictidae | RSA | Below | pl | solitary | 3.07 | Scopa_leg | 4.03 |  | 8 |
| *Pseudoanthidium* sp1_10 | Megachilidae | RSA | Above |  | solitary | 2.85 | Scopa_abdomen | 5.11 |  | 1 |
| *Pseudoanthidium tuberculiferum* FRIESE 1905 | Megachilidae | RSA | Above | pl | solitary | 3.31 | Scopa_abdomen | 5.90 | spring_summer | 3 |
| **Scrapter heterodoxus* (COCKERELL 1921) | Colletidae | RSA | Below | pl | solitary | 2.93 | Scopa_leg | 2.43 | spring | 4 |
| *Tetraloniella junodi* (FRIESE 1909) | Apidae | RSA | Below | pl | solitary | 2.92 | Scopa_leg | 5.97 | whole veg. Period | 65 |
| *Xylocopa rufitarsis* LEPELETIER 1841 | Apidae | RSA | Above | pl | solitary | 3.97 | Scopa_leg | 8.03 | whole veg. Period | 2 |

Note: *: Species endemic to South Africa; AT: Austria, RSA: South Africa; Above: Above-ground nesting species use pre-existing cavities for nesting, Below: Below- ground nesting species excavating the nests into the ground; pl: polylectic, ol: oligolectic; Pollen collection type: Part of the body where pollen is stored for transport: Corbicula; Scopa_leg=Tibial scopa; Abdominal scopa; Crop (ingested); Seasonality: Activity during vegetation period: Spring (AT: III – V, SA: IX – XI); Early summer (AT: V – VI, SA: XI – XII); Summer (AT: VI – VIII, SA: XII – II); Late summer (AT: IX, SA: III); Whole vegetation period (AT: III – X, SA: IX – IV); ITD: Inter-tegular distance (mm)= The shortest linear distance measured between wing tegulae across the dorsal thorax (Cane, 1987);

Table S2: Flowering plant species list including family and frequency (amount of vineyard the species occurred in). Information of functional traits as derived from literature and the TRY data base (Kattge *et al.*, 2020; detailed references are listed in Section "Appendix-TRY references")and how they were aggregated (simplified) for data analysis (calculation of functional richness and community weighted means).

| **Species** | **Family** | **Frequ-ency** | **Flower morphology** | | **Nectar accessibility** | | **Nectar presence** | **Flower colour** | | **Flower symmetry** | | **Seasonality** | |
| --- | --- | --- | --- | --- | --- | --- | --- | --- | --- | --- | --- | --- | --- |
|  |  |  | Original (Kugler, 1970) | Simplified | Original (Müller, 1881; Kugler, 1970) | Simplified |  | Original (TRY) | Simplified | Original (TRY) | Simplified | Months | Season |
| **Austria & South Africa** | | | | | | | | | | | | | |
| *Plantago lanceolata* | Plantaginaceae | 22 | wind flowers occassionally visited by insect | wind_pollen | wind flowers occassionally visited by insect | wind_pollen | nectar none | white_brown | white | radial | radial | V-IX_X-III | summer |
| **Austria** | | | | | | | | | | | | | |
| *Achillea millefolium* | Asteraceae | 22 | flower heads, Asteraceae, ray and disk flowers | flower heads, ray and disk | flower associations with totally hidden nectar | flower associations with totally hidden nectar | nectar present | white | white | radial | radial | VI-XI | summer |
| *Ajuga genevensis* | Lamiaceae | 1 | true lip flowers | lip flowers | hymenopteran flowers | bee flower | nectar present | blue | blue | bilateral | bilateral | IV-VI | spring |
| *Anthemis arvensis* | Asteraceae | 1 | flower heads, Asteraceae, ray and disk flowers | flower heads, ray and disk | flower associations with totally hidden nectar | flower associations with totally hidden nectar | nectar present | white_yellow | white_yellow | radial | radial | VI-IX | summer |
| *Anthriscus sylvestris* | Apiaceae | 2 | disk flowers with nectar open | disk flowers | nectar open | nectar open | nectar present | white | white | biradial | radial | VI-VIII | summer |
| *Bellis perennis* | Asteraceae | 1 | flower heads, Asteraceae, ray and disk flowers | flower heads, ray and disk | flower associations with totally hidden nectar | flower associations with totally hidden nectar | nectar present | white_yellow | white_yellow | radial | radial | III-X | wholeYear |
| *Brassica napus* | Brassicaceae | 2 | disk flowers with nectar ± hidden nectaries at base of stamens | disk flowers | nectar ± hidden | nectar ± hidden | nectar present | yellow | yellow | radial | radial | IV-IX | early summer |
| *Calendula officinalis* | Asteraceae | 1 | flower heads, Asteraceae, ray and disk flowers | flower heads, ray and disk | flower associations with totally hidden nectar | flower associations with totally hidden nectar | nectar present | orange | red_orange | radial | radial | VII-IX | late summer |
| *Camelia sativa* | Brassicaceae | 1 | disk flowers with nectar ± hidden nectaries at base of stamens | disk flowers | nectar ± hidden | nectar ± hidden | nectar present | yellow | yellow | radial | radial | V-VIII | early summer |
| *Capsella bursa-pastoris* | Brassicaceae | 18 | disk flowers with nectar ± hidden nectaries at base of stamens | disk flowers | nectar ± hidden | nectar ± hidden | nectar present | white | white | biradial | radial | I-XII | wholeYear |
| *Cardaria draba* | Brassicaceae | 1 | disk flowers with nectar ± hidden nectaries at base of stamens | disk flowers | nectar ± hidden | nectar ± hidden | nectar present | white | white | radial | radial | IV-VI | spring |
| *Carduus acantuides* | Asteraceae | 4 | flower heads, Asteraceae, only ray flowers | flower heads, only ray flowers | flower associations with totally hidden nectar | flower associations with totally hidden nectar | nectar present | purple | purple | radial | radial | VI-X | summer |
| *Carum carvi* | Apiaceae | 1 | disk flowers with nectar open | disk flowers | nectar open | nectar open | nectar present | white | white | radial | radial | V-VII | early summer |
| *Centaurea cyanus* | Asteraceae | 3 | flower heads, Asteraceae, only ray flowers | flower heads, only ray flowers | flower associations with totally hidden nectar | flower associations with totally hidden nectar | nectar present | blue | blue | radial | radial | VI-X | summer |
| *Cerastium holosteoides* | Caryophyllaceae | 1 | disk flowers with nectar ± hidden nectaries at base of stamens | disk flowers | nectar ± hidden | nectar ± hidden | nectar present | white | white | radial | radial | III-VI | spring |
| *Cerastium* sp. | Caryophyllaceae | 3 | disk flowers with nectar ± hidden nectaries at base of stamens | disk flowers | nectar ± hidden | nectar ± hidden | nectar present | white | white | radial | radial | NA | NA |
| *Cichorium intybus* | Asteraceae | 3 | flower heads, Asteraceae, only ray flowers | flower heads, only ray flowers | flower associations with totally hidden nectar | flower associations with totally hidden nectar | nectar present | blue | blue | radial | radial | VII | summer |
| *Cirsium arvense* | Asteraceae | 1 | flower heads, Asteraceae, only disc flowers | flower heads, only disc flowers | flower associations with totally hidden nectar | flower associations with totally hidden nectar | nectar present | pink | pink | radial | radial | VII-IX | late summer |
| *Consolida regalis* | Ranunculaceae | 2 | lip flowers, throat flowers | lip flowers | throat flowers, bumble bee flower | bee flower | nectar present | blue | blue | bilateral | bilateral | V-VIII | early summer |
| *Convulvulus arvensis* | Convolvulaceae | 24 | funnel flowers, large | bell_funnel | flower associations with totally hidden nectar | flower associations with totally hidden nectar | nectar present | white_pink | pink | radial | radial | VI-IX | summer |
| *Crepis foetida* | Asteraceae | 7 | flower heads, Asteraceae, only ray flowers | flower heads, only ray flowers | flower associations with totally hidden nectar | flower associations with totally hidden nectar | nectar present | yellow | yellow | radial | radial | VI-VIII | summer |
| *Crepis setosa* | Asteraceae | 1 | flower heads, Asteraceae, only ray flowers | flower heads, only ray flowers | flower associations with totally hidden nectar | flower associations with totally hidden nectar | nectar present | yellow | yellow | radial | radial | VI-VIII | summer |
| *Crepis* sp*.* | Asteraceae | 1 | flower heads, Asteraceae, only ray flowers | flower heads, only ray flowers | flower associations with totally hidden nectar | flower associations with totally hidden nectar | nectar present | yellow | yellow | radial | radial | NA | NA |
| *Daucus carota* | Apiaceae | 8 | disk flowers with nectar open | disk flowers | nectar open | nectar open | nectar present | white | white | radial, bilateral at the border | radial | VI-IX | summer |
| *Erigeron annuus* | Asteraceae | 17 | flower heads, Asteraceae, ray and disk flowers | flower heads, ray and disk | flower associations with totally hidden nectar | flower associations with totally hidden nectar | nectar present | white_yellow | white_yellow | radial | radial | VI-IX | summer |
| *Erigeron canadensis* | Asteraceae | 9 | flower heads, Asteraceae, only disc flowers | flower heads, only disc flowers | flower associations with totally hidden nectar | flower associations with totally hidden nectar | nectar present | white_yellow | white_yellow | radial | radial | VII-IX | late summer |
| *Erodium cicutarium* | Geraniaceae | 4 | disk flowers with nectar ± hidden nectaries at base of stamens | disk flowers | nectar ± hidden | nectar ± hidden | nectar present | purple | purple | radial | radial | III-XI | wholeYear |
| *Fagopyrum esculentum* | Polygonaceae | 5 | disk flowers with nectar open | disk flowers | nectar open | nectar open | nectar present | white_pink | pink | radial | radial | VI-IX | summer |
| *Falcaria vulgaris* | Apiaceae | 2 | disk flowers with nectar open | disk flowers | nectar open | nectar open | nectar present | white | white | radial | radial | VII-IX | late summer |
| *Geranium pusillum* | Geraniaceae | 15 | disk flowers with nectar ± hidden nectaries at base of stamens | disk flowers | nectar ± hidden | nectar ± hidden | nectar present | lila | pink | radial | radial | V-X | early summer |
| *Hypericum perforatum* | Hypericaceae | 1 | pollen flowers | disk flowers | pollen flower | wind_pollen | nectar none | yellow | yellow | radial | radial | VI-VIII | summer |
| *Lactuca serriola* | Asteraceae | 1 | flower heads, Asteraceae, only ray flowers | flower heads, only ray flowers | flower associations with totally hidden nectar | flower associations with totally hidden nectar | nectar present | yellow | yellow | radial | radial | VII-IX | late summer |
| *Lamium amplexicaule* | Lamiaceae | 2 | true lip flowers | lip flowers | hymenopteran flowers | bee flower | nectar present | purple | purple | bilateral | bilateral | IV-V | spring |
| *Lamium purpureum* | Lamiaceae | 23 | true lip flowers | lip flowers | hymenopteran flowers | bee flower | nectar present | purple | purple | bilateral | bilateral | III-X | wholeYear |
| *Lathyrus sativus* | Fabaceae | 1 | flag blossom, Fabaceae type, explosive mechanism | flag blossom | Fabaceae type | Fabaceae type | nectar present | white | white | bilateral | bilateral | V-VI | early summer |
| *Lotus corniculatus* | Fabaceae | 4 | flag blossom, Fabaceae type, brush mechanism | flag blossom | Fabaceae type | Fabaceae type | nectar present | yellow | yellow | bilateral | bilateral | VI-VIII | summer |
| *Malva moschata* | Malvaceae | 3 | disk flowers with nectar ± hidden nectaries at base of stamens | disk flowers | nectar ± hidden | nectar ± hidden | nectar present | white_pink | pink | radial | radial | VI-X | summer |
| *Malva neglecta* | Malvaceae | 3 | disk flowers with nectar ± hidden nectaries at base of stamens | disk flowers | nectar ± hidden | nectar ± hidden | nectar present | white_pink | pink | radial | radial | VI-X | summer |
| *Matricaria discoidea* | Asteraceae | 1 | flower heads, Asteraceae, only disc flowers | flower heads, only disc flowers | flower associations with totally hidden nectar | flower associations with totally hidden nectar | nectar present | yellow_green | yellow | radial | radial | V-IX | early summer |
| *Medicago lupulina* | Fabaceae | 17 | flag blossom, Fabaceae type, explosive mechanism | flag blossom | Fabaceae type | Fabaceae type | nectar present | yellow | yellow | bilateral | bilateral | V-X | early summer |
| *Medicago sativa* | Fabaceae | 8 | flag blossom, Fabaceae type, explosive mechanism | flag blossom | Fabaceae type | Fabaceae type | nectar present | purple | purple | bilateral | bilateral | V-X | early summer |
| *Melilotus albus* | Fabaceae | 1 | flag blossom, Fabaceae type, valvular mechanism | flag blossom | Fabaceae type | Fabaceae type | nectar present | white | white | bilateral | bilateral | V-VIII | early summer |
| *Melilotus officinalis* | Fabaceae | 6 | flag blossom, Fabaceae type, valvular mechanism | flag blossom | Fabaceae type | Fabaceae type | nectar present | yellow | yellow | bilateral | bilateral | V-IX | early summer |
| *Muscari neglectum* | Liliaceae/Asparagaceae | 2 | bell shaped flowers with sticky pollen | bell_funnel | hymenopteran flowers | bee flower | nectar present | darkblue | blue | radial | radial | IV-V | spring |
| *Myosotis arvensis* | Boraginaceae | 3 | stalk disc flowers, stamina and pistil within tube | disk flowers | stamina and pistil within tube | flowers with totally hidden nectar | nectar present | blue_yellow | blue | radial | radial | IV-IX | early summer |
| *Nonea pulla* | Boraginaceae | 1 | bell shaped flowers with powdery pollen | bell_funnel | hymenopteran flowers | bee flower | nectar present | darkpurple | purple | radial | radial | V-VIII | early summer |
| *Onobrychis viciifolia* | Fabaceae | 7 | flag blossom, Fabaceae type, valvular mechanism | flag blossom | Fabaceae type | Fabaceae type | nectar present | pink | pink | bilateral | bilateral | V-VII | early summer |
| *Papaver rhoeas* | Papaveraceae | 4 | pollen flowers | disk flowers | pollen flower | wind_pollen | nectar none | red | red_orange | radial | radial | V-VII | early summer |
| *Phacelia tanacetifolia* | Boraginaceae | 8 | funnel flowers, small | bell_funnel | hymenopteran flowers | bee flower | nectar present | blue | blue | radial | radial | VI-VII | summer |
| *Picris hieracioides* | Asteraceae | 1 | flower heads, Asteraceae, only ray flowers | flower heads, only ray flowers | flower associations with totally hidden nectar | flower associations with totally hidden nectar | nectar present | yellow | yellow | radial | radial | VI-X | summer |
| *Polygonum aviculare* | Polygonaceae | 3 | bell shaped flowers with sticky pollen | bell_funnel | flowers with totally hidden nectar | flowers with totally hidden nectar | nectar present | pink | pink | radial | radial | VI-X | summer |
| *Prunella vulgaris* | Lamiaceae | 2 | true lip flowers | lip flowers | hymenopteran flowers | bee flower | nectar present | violet | purple | bilateral | bilateral | VI-IX | summer |
| *Ranunculus bulbosus* | Ranunculaceae | 1 | disk flowers with nectar ± hidden nectaries at base of stamens | disk flowers | nectar ± hidden | nectar ± hidden | nectar present | yellow | yellow | radial | radial | IV-V | spring |
| *Raphanus sativus* | Brassicaeae | 5 | disk flowers with nectar open | disk flowers | nectar open | nectar open | nectar present | white | white | radial | radial | V-VI | early summer |
| *Sanguisorba minor* | Rosaceae | 4 | pollen flowers | wind_pollen | wind flowers | wind_pollen | nectar none | red | red_orange | radial | radial | VI-VII | summer |
| *Senecio vernalis* | Asteraceae | 1 | flower heads, Asteraceae, ray and disk flowers | flower heads, ray and disk | flower associations with totally hidden nectar | flower associations with totally hidden nectar | nectar present | yellow | yellow | radial | radial | IV-V | spring |
| *Sisymbrium loeselii* | Brassicaeae | 1 | disk flowers with nectar ± hidden nectaries at base of stamens | disk flowers | nectar ± hidden | nectar ± hidden | nectar present | yellow | yellow | radial | radial | IV-XI | wholeYear |
| *Sonchus asper* | Asteraceae | 1 | flower heads, Asteraceae, only ray flowers | flower heads, only ray flowers | flower associations with totally hidden nectar | flower associations with totally hidden nectar | nectar present | yellow | yellow | radial | radial | VI-X | summer |
| *Stellaria media* | Caryophyllaceae | 24 | disk flowers with nectar ± hidden nectaries at base of stamens | disk flowers | nectar ± hidden | nectar ± hidden | nectar present | white | white | radial | radial | I-XII | wholeYear |
| *Taraxacum officinale* | Asteraceae | 24 | flower heads, Asteraceae, only ray flowers | flower heads, only ray flowers | flower associations with totally hidden nectar | flower associations with totally hidden nectar | nectar present | yellow | yellow | radial | radial | IV-V | spring |
| *Tragopogon* sp. | Asteraceae | 2 | flower heads, Asteraceae, only ray flowers | flower heads, only ray flowers | flower associations with totally hidden nectar | flower associations with totally hidden nectar | nectar present | yellow | yellow | radial | radial | V-VI | early summer |
| *Trifolium campestre* | Fabaceae | 4 | flag blossom, Fabaceae type, valvular mechanism | flag blossom | Fabaceae type | Fabaceae type | nectar present | yellow | yellow | bilateral | bilateral | VI-X | summer |
| *Trifolium hybridum* | Fabaceae | 1 | flag blossom, Fabaceae type, valvular mechanism | flag blossom | Fabaceae type | Fabaceae type | nectar present | white_pink | pink | bilateral | bilateral | V-IX | early summer |
| *Trifolium incarnatum* | Fabaceae | 11 | flag blossom, Fabaceae type, valvular mechanism | flag blossom | Fabaceae type | Fabaceae type | nectar present | red | red_orange | bilateral | bilateral | V-VIII | early summer |
| *Trifolium pannonicum* | Fabaceae | 1 | flag blossom, Fabaceae type, valvular mechanism | flag blossom | Fabaceae type | Fabaceae type | nectar present | white | white | bilateral | bilateral | VI | summer |
| *Trifolium pratense* | Fabaceae | 12 | flag blossom, Fabaceae type, valvular mechanism | flag blossom | Fabaceae type | Fabaceae type | nectar present | red | pink | bilateral | bilateral | V-IX | early summer |
| *Trifolium repens* | Fabaceae | 20 | flag blossom, Fabaceae type, valvular mechanism | flag blossom | Fabaceae type | Fabaceae type | nectar present | white | white | bilateral | bilateral | V-X | early summer |
| *Trifolium resupinatum* | Fabaceae | 1 | flag blossom, Fabaceae type, valvular mechanism | flag blossom | Fabaceae type | Fabaceae type | nectar present | pink | pink | bilateral | bilateral | V-VII | early summer |
| *Tripleurospermum inodorum* | Asteraceae | 2 | flower heads, Asteraceae, ray and disk flowers | flower heads, ray and disk | flower associations with totally hidden nectar | flower associations with totally hidden nectar | nectar present | white_yellow | white_yellow | radial | radial | VI-XI | summer |
| *Valerianella carinata* | Caprifoliaceae | 4 | funnel flowers, small | bell_funnel | flowers with totally hidden nectar | flowers with totally hidden nectar | nectar present | lightblue | blue | bilateral | bilateral | IV-V | spring |
| *Verbena officinalis* | Verbenaceae | 1 | funnel flowers, small | bell_funnel | hymenopteran flowers | bee flower | nectar present | violet | purple | bilateral | bilateral | VII-IX | late summer |
| *Veronica persica* | Plantaginaceae/Veronicaceae | 24 | lip flowers, Verbascum type | lip flowers | flowers with totally hidden nectar | flowers with totally hidden nectar | nectar present | lightblue | blue | bilateral | bilateral | I-XII | wholeYear |
| *Veronica sp.1* | Plantaginaceae/Veronicaceae | 2 | lip flowers, Verbascum type | lip flowers | flowers with totally hidden nectar | flowers with totally hidden nectar | nectar present | lightblue | blue | bilateral | bilateral | NA | NA |
| *Veronica sp.2* | Plantaginaceae/Veronicaceae | 4 | lip flowers, Verbascum type | lip flowers | flowers with totally hidden nectar | flowers with totally hidden nectar | nectar present | lightblue | blue | bilateral | bilateral | NA | NA |
| *Veronica sp.3 (very small)* | Plantaginaceae/Veronicaceae | 1 | lip flowers, Verbascum type | lip flowers | flowers with totally hidden nectar | flowers with totally hidden nectar | nectar present | lightblue | blue | bilateral | bilateral | NA | NA |
| *Vicia angustifolia* | Fabaceae | 8 | flag blossom, Fabaceae type, explosive mechanism | flag blossom | Fabaceae type | Fabaceae type | nectar present | purple | purple | bilateral | bilateral | V-VI | early summer |
| *Vicia faba* | Fabaceae | 2 | flag blossom, Fabaceae type, explosive mechanism | flag blossom | Fabaceae type | Fabaceae type | nectar present | white | white | bilateral | bilateral | V-VII | early summer |
| *Vicia hirsuta* | Fabaceae | 1 | flag blossom, Fabaceae type, explosive mechanism | flag blossom | Fabaceae type | Fabaceae type | nectar present | white | white | bilateral | bilateral | V-VII | early summer |
| *Vicia pannonica* | Fabaceae | 7 | flag blossom, Fabaceae type, explosive mechanism | flag blossom | Fabaceae type | Fabaceae type | nectar present | white | white | bilateral | bilateral | V-VII | early summer |
| *Viola arvensis* | Violaceae | 4 | lip flowers, Viola type | lip flowers | bee flowers | bee flower | nectar present | white | white | bilateral | bilateral | IV-X | wholeYear |
| **South Africa** | | | | | | | | | | | | | |
| *Arctotis acaulus* | Asteraceae | 2 | flower heads, Asteraceae, ray and disk flowers | flower heads, ray and disk | flower associations with totally hidden nectar | flower associations with totally hidden nectar | nectar present | yellow_orange | yellow | radial | radial | VIII-X | spring |
| *Echium plantagineum* | Boraginaceae | 6 | lip flowers, throat flowers | lip flowers | throat flowers, bee flowers | bee flower | nectar present | purple | purple | bilateral | bilateral | X-IV | wholeYear |
| *Helichrysum cymosum* | Asteraceae | 1 | flower heads, Asteraceae, only disc flowers | flower heads, only disc flowers | flower associations with totally hidden nectar | flower associations with totally hidden nectar | nectar present | yellow | yellow | radial | radial | IX-IV | wholeYear |
| *Helichrysum grandiflorum* | Asteraceae | 1 | flower heads, Asteraceae, only disc flowers | flower heads, only disc flowers | flower associations with totally hidden nectar | flower associations with totally hidden nectar | nectar present | white_yellow | white_yellow | radial | radial | XII-II | early summer |
| *Indigofera* | Fabaceae | 2 | flag blossom, Fabaceae type, explosive mechanism | flag blossom | Fabaceae type | Fabaceae type | nectar present | purple | purple | bilateral | bilateral | NA | NA |
| *Medicago polymorpha* | Fabaceae | 1 | flag blossom, Fabaceae type, explosive mechanism | flag blossom | Fabaceae type | Fabaceae type | nectar present | yellow | yellow | bilateral | bilateral | VI-X | spring |
| *Picris echioides (Helminthotheca echioides)* | Asteraceae | 3 | flower heads, Asteraceae, only ray flowers | flower heads, only ray flowers | flower associations with totally hidden nectar | flower associations with totally hidden nectar | nectar present | yellow | yellow | radial | radial | IX-X | spring |
| *Raphanus raphanistrum* | Brassicaeae | 9 | disk flowers with nectar ± hidden nectaries at base of stamens | disk flowers | nectar ± hidden | nectar ± hidden | nectar present | white_purple | pink | radial | radial | VI-XII | spring |
| *Senecio burchelli* | Asteraceae | 5 | flower heads, Asteraceae, ray and disk flowers | flower heads, ray and disk | flower associations with totally hidden nectar | flower associations with totally hidden nectar | nectar present | yellow | yellow | radial | radial | IV-VI | autumn |
| *Senecio pterophorus* | Asteraceae | 1 | flower heads, Asteraceae, ray and disk flowers | flower heads, ray and disk | flower associations with totally hidden nectar | flower associations with totally hidden nectar | nectar present | yellow | yellow | radial | radial | X-I | spring |
| *Senecio* sp*.* | Asteraceae | 9 | flower heads, Asteraceae, ray and disk flowers | flower heads, ray and disk | flower associations with totally hidden nectar | flower associations with totally hidden nectar | nectar present | yellow | yellow | radial | radial | NA | NA |
| *Trifolium angustifolium* | Fabaceae | 3 | flag blossom, Fabaceae type, valvular mechanism | flag blossom | Fabaceae type | Fabaceae type | nectar present | pink | pink | bilateral | bilateral | IX-XI | spring |
| *Ursinia* sp. | Asteraceae | 1 | flower heads, Asteraceae, ray and disk flowers | flower heads, ray and disk | flower associations with totally hidden nectar | flower associations with totally hidden nectar | nectar present | yellow | yellow | radial | radial | NA | NA |
| *Vicia benghalensis* | Fabaceae | 7 | flag blossom, Fabaceae type, explosive mechanism | flag blossom | Fabaceae type | Fabaceae type | nectar present | purple | purple | bilateral | bilateral | X-XI | spring |

Note: *Flower morphology*: Flowers classified according to shape: Bell & funnel flowers; Disk flowers; Flag blossom; Asteraceae, only disc flower heads; Asteraceae, only ray flower heads; Asteraceae, ray & disc flower heads; Lip flowers; Wind pollinated & pollen flowers. *Nectar accessibility:* Classification according to location of reward: Bee flower; Fabaceae type; Nectar ± hidden; Flower associations with totally hidden nectar; Flowers with totally hidden nectar; Nectar openly available; Wind pollinated & pollen flowers. *Nectar presence*: Flowers with Nectar present or No nectar present. *Flower symmentry*: Flowers with Radial or Bilateral symmetry. *Flower colour*: Different shades of the following colours were grouped: Blue; Pink; Purple; Red & Orange; White; White & Yellow; Yellow. *Seasonality:* Flowering during vegetation period: Spring (AT: III – V, SA: IX – XI); Early summer (AT: V – VI, SA: XI – XII); Summer (AT: VI – VIII, SA: XII – II); Late summer (AT: IX, SA: III); Whole vegetation period (AT: III – X, SA: IX – IV)

Table S3: Details of the most parsimonious models for bee and plant functional richness and bee species richness in Austrian and South African vineyard inter-rows. General information for models in bold.

| **Response variable** | **Model and details for explanatory variable** | **Estimate ± SE** | **General model information** | | |
| --- | --- | --- | --- | --- | --- |
|  |  |  | **AICc** | **R²m** | **R²c** |
| **Wild bee FRic** | **~ flowering plant FRic : farm type** |  | **86.65** | **0.47** | **0.47** |
|  | flowering plant FRic:farm_organic | 0.945 ± 0.191 |  |  |  |
|  | flowering plant FRic:farm_conv. | 0.393 ± 0.162 |  |  |  |
|  | **~ flowering plant FRic** | 0.624 ± 0.130 | **88.41** | **0.40** | **0.40** |
|  | **~ flowering plant FRic + woody structures** |  | **88.63** | **0.45** | **0.45** |
|  | flowering plant FRic | 0.586 ± 0.128 |  |  |  |
|  | woody structures | 0.207 ± 0.128 |  |  |  |
| **Wild bee SPRic** | **~ flowering plant FRic + inter-row veg. cover** |  | **95.75** | **0.31** | **0.43** |
|  | flowering plant FRic | 0.519 ± 0.145 |  |  |  |
|  | inter-row veg. cover | 0.280 ± 0.140 |  |  |  |
|  | **~ flowering plant FRic** | 0.511 ± 0.151 | 96.32 | 0.25 | 0.51 |
|  | **~ flowering plant FRic + woody structures** |  | 96.86 | 0.30 | 0.35 |
|  | flowering plant FRic | 0.423 ± 0.146 |  |  |  |
|  | woody structure | 0.275 ± 0.146 |  |  |  |
|  | **~ flowering plant FRic + SNHs** |  | 97.65 | 0.30 | **0.52** |
|  | flowering plant FRic | 0.487 ± 0.150 |  |  |  |
|  | SNHs | -0.198 ± 0.164 |  |  |  |
| **Flowering plant FRic** | ~ **farm type : wild bee FRic** |  | **90.31** | **0.42** | **0.43** |
|  | farm_organic:wild bee FRic | 0.829 ± 0.179 |  |  |  |
|  | farm_conv:wild bee FRic | 0.404 ± 0.174 |  |  |  |
|  | **~ wild bee FRic** |  | **90.87** | **0.37** | **0.37** |
|  | **~ inter-row veg. cover + wild bee FRic** | 0.614 ± 0.134 | **91.71** | **0.40** | **0.40** |
|  | inter-row veg. cover | -0.015 ± 0.128 |  |  |  |
|  | Wild bee FRic | 0.646 ± 0.129 |  |  |  |

Note: *FRic*: Functional richness; *SPRic*: Species richness; *SE*: Standard Error, *AICc* = Akaike Information Criterion; *R²m* = marginal R²; *R²c* = conditional R²

Table S4: CWMs of bees and plants per vineyard, including information about farm type, flowering plant and wild bee functional richness and proportions of semi-natural habitats and woodlots within 500 m radius around each vineyard.

| **Vineyard ID** | **Landscape ID** | **Farm**  **type** | **FRic Plants** | **CWM flowering plants** | | | | | **FRic Wild bees** | **CWM wild bees** | | | | | | | **Landscape** | |
| --- | --- | --- | --- | --- | --- | --- | --- | --- | --- | --- | --- | --- | --- | --- | --- | --- | --- | --- |
|  |  |  |  | **Seasonality** | **Flower morph.** | **Nectar accessibility** | **Flower colour** | **Flower symmetry** |  | **Lecty** | **Nesting type** | **Sociality** | **ITD (mm)** | **Pollen transport** | **Proboscis (mm)** | **Seasonality** | **SNH %** | **Woodlot %** |
| **Austria** | | | | | | | | | | | | | | | | | | |
| A_01_HI | Ale01 | convent | 14.00 | wholeYear | disk | fl.assoc_thn | white | radial | 0.12 | pl | Below | solitary | 1.64 | Scopa_leg | 2.17 | whole veg. period | 23.07 | 1.32 |
| A_02_HI | Ale02 | convent | 22.00 | summer | flag | fl.assoc_thn | white | radial | 1.39 | pl | Above | eusocial | 2.87 | Corbicula | 5.28 | whole veg. period | 21.45 | 1.73 |
| A_02_LO | Ale02 | convent | 18.00 | summer | flag | fl.assoc_thn | white | radial | 0.87 | pl | Below | solitary | 1.79 | Scopa_leg | 2.95 | whole veg. period | 21.45 | 1.73 |
| A_03_HI | Ale03 | convent | 16.00 | early_su | flag | faba | white | bilateral | 1.61 | pl | Below | solitary | 2.59 | Scopa_leg | 4.71 | whole veg. period | 27.42 | 9.10 |
| A_04_HI | Ale04 | organic | 17.00 | summer | disk | fl.assoc_thn | white | radial | 1.25 | pl | Below | eusocial | 2.05 | Scopa_leg | 2.96 | spring | 36.98 | 32.81 |
| A_04_LO | Ale04 | convent | 14.00 | summer | disk | fl.assoc_thn | white | radial | 1.37 | pl | Above | eusocial | 2.57 | Scopa_leg | 4.51 | whole veg. period | 36.98 | 32.81 |
| A_05_HI | Ale05 | organic | 20.00 | early_su | flag | faba | pink | radial | 1.19 | pl | Below | solitary | 2.24 | Scopa_leg | 3.73 | whole veg. period | 10.97 | 1.31 |
| A_05_LO | Ale05 | convent | 17.00 | summer | disk flowers | n+-h | white | radial | 1.25 | pl | Below | solitary | 1.90 | Scopa_leg | 2.62 | summer | 10.97 | 1.31 |
| A_06_LO | Ale06 | organic | 18.00 | early_su | flag | faba | white | bilateral | 1.71 | pl | Below | solitary | 2.45 | Scopa_leg | 3.74 | spring_summer | 14.42 | 3.36 |
| A_07_HI | Ale07 | organic | 25.00 | summer | flag | fl.assoc_thn | white | radial | 2.19 | pl | Below | eusocial | 2.31 | Scopa_leg | 3.96 | whole veg. period | 11.57 | 51.49 |
| A_07_LO | Ale07 | organic | 19.00 | early_su | flag | faba | white | radial | 0.82 | pl | Below | eusocial | 1.84 | Scopa_leg | 2.77 | whole veg. period | 11.57 | 51.49 |
| A_08_LO | Ale08 | organic | 17.00 | summer | bell | fl.assoc_thn | purple | radial | 0.96 | pl | Below | solitary | 1.96 | Scopa_leg | 3.15 | whole veg. period | 25.94 | 13.46 |
| A_09_HI | Aca09 | convent | 15.00 | summer | flag | faba | white | radial | 0.49 | pl | Below | eusocial | 1.81 | Scopa_leg | 2.59 | whole veg. period | 2.11 | 2.66 |
| A_09_LO | Aca09 | convent | 15.00 | summer | h_ray | fl.assoc_thn | yellow | radial | 0.98 | pl | Below | eusocial | 1.80 | Scopa_leg | 2.54 | whole veg. period | 2.11 | 2.66 |
| A_10_HI | Aca10 | convent | 19.00 | early_su | flag | faba | white | radial | 0.38 | pl | Below | eusocial | 2.08 | Scopa_leg | 3.18 | spring | 7.59 | 14.99 |
| A_10_LO | Aca10 | convent | 12.00 | wholeYear | flag | faba | white | bilateral | 0.62 | pl | Below | eusocial | 2.00 | Scopa_leg | 2.91 | spring | 7.59 | 14.99 |
| A_11_HI | Aca11 | convent | 14.00 | early_su | flag | faba | pink | bilateral | 1.71 | pl | Below | eusocial | 2.01 | Scopa_leg | 3.00 | spring | 9.16 | 48.87 |
| A_11_LO | Aca11 | convent | 17.00 | summer | disk | fl.assoc_thn | white | radial | 0.63 | pl | Below | eusocial | 1.80 | Scopa_leg | 2.49 | spring | 9.16 | 48.87 |
| A_12_LO | Aca12 | convent | 17.00 | summer | flag | fl.assoc_thn | yellow | radial | 0.99 | pl | Below | eusocial | 1.84 | Scopa_leg | 2.37 | spring | 4.68 | 18.17 |
| A_13_HI | Aca13 | convent | 19.00 | early_su | flag | faba | white | radial | 0.33 | pl | Below | eusocial | 2.61 | Corbicula | 4.60 | whole veg. period | 12.03 | 0.60 |
| A_14_HI | Aca14 | convent | 17.00 | early_su | flag | faba | white | bilateral | 0.94 | pl | Below | solitary | 1.77 | Scopa_leg | 2.71 | whole veg. period | 12.96 | 1.05 |
| A_15_LO | Aca15 | convent | 17.00 | summer | flag | fl.assoc_thn | white | radial | 1.35 | pl | Below | eusocial | 1.88 | Scopa_leg | 2.70 | spring | 38.79 | 17.68 |
| A_16_HI | Aca16 | organic | 13.00 | summer | disk | fl.assoc_thn | white | radial | 0.30 | pl | Below | solitary | 2.09 | Scopa_leg | 3.03 | whole veg. period | 45.73 | 4.60 |
| A_16_only2015 | Aca16 | convent | 8.00 | wholeYear | disk | n+-h | white | radial | 0.01 | pl | Below | eusocial | 2.50 | Scopa_leg | 4.22 | spring | 45.73 | 4.60 |
| A_16_only2016 | Aca16 | convent | 13.00 | summer | bell | fl.assoc_thn | blue | radial | 0.43 | pl | Below | eusocial | 1.83 | Scopa_leg | 2.77 | whole veg. period | 45.73 | 4.60 |
| **South Africa** | | | | | | | | | | | | | | | | | | |
| Firgrove_C | Firgrove | convent | 7.00 | spring | h_raydisk | fl.assoc_thn | yellow | radial | 0.45 | pl | Below | solitary | 2.19 | Scopa_leg | 2.80 | whole veg. period | 10.07 | 5.08 |
| Firgrove_O | Firgrove | organic | 4.00 | spring | h_raydisk | fl.assoc_thn | yellow | radial | 0.13 | pl | Below | solitary | 2.20 | Scopa_leg | 2.82 | whole veg. period | 10.32 | 5.22 |
| Joostenberg_C | Joostenberg | convent | 5.00 | spring | h_raydisk | fl.assoc_thn | purple | radial | 0.31 | pl | Below | solitary | 2.39 | Scopa_leg | 2.79 | whole veg. period | 37.45 | 4.73 |
| Joostenberg_O | Joostenberg | organic | 5.00 | spring | h_raydisk | fl.assoc_thn | yellow | radial | 0.11 | pl | Below | solitary | 2.54 | Scopa_leg | 3.70 | whole veg. period | 58.24 | 8.21 |
| Laibach_C | Laibach | convent | 5.00 | spring | disk | fl.assoc_thn | yellow | radial | 0.42 | pl | Below | solitary | 1.91 | Scopa_leg | 2.62 | whole veg. period | 26.74 | 6.59 |
| Laibach_O | Laibach | organic | 5.00 | autumn | h_raydisk | fl.assoc_thn | yellow | radial | 0.29 | pl | Below | solitary | 2.58 | Scopa_leg | 3.79 | whole veg. period | 13.13 | 5.80 |
| Spier_C | Spier | convent | 6.00 | spring | h_raydisk | fl.assoc_thn | yellow | radial | 0.31 | pl | Below | solitary | 2.33 | Scopa_leg | 2.92 | whole veg. period | 26.90 | 2.01 |
| Spier_O | Spier | organic | 7.00 | spring | flag | faba | purple | bilateral | 0.56 | pl | Below | solitary | 2.26 | Scopa_leg | 2.96 | whole veg. period | 72.53 | 7.66 |
| Uitzicht Conventional | Uitzicht | convent | 4.00 | spring | flag | faba | yellow | radial | 0.04 | pl | Below | solitary | 2.60 | Scopa_leg | 2.75 | whole veg. period | 12.73 | 5.86 |
| Uitzicht Organic | Uitzicht | organic | 4.00 | spring | disk | n+-h | purple | radial | 0.04 | pl | Below | solitary | 2.58 | Scopa_leg | 2.76 | whole veg. period | 14.42 | 3.83 |

Note: Abbreviations: Flower morphology: bell: Bell & funnel flowers; disk: Disk flowers; flag: Flag blossom; h_disk: Asteraceae, only disc flower heads; h_ray: Asteraceae, only ray flower heads; h_raydisk: Asteraceae, ray & disc flower heads; lip: Lip flowers; Nectar accessibility: faba: Fabaceae type; n+-h: Nectar ± hidden; fl.assoc_thn: Flower associations with totally hidden nectar; thn: Flowers with totally hidden nectar; pl: polylectic, Above: Above-ground nesting species, Below: Below- ground nesting species excavating the nests into the ground; Pollen collection type: Part of the body where pollen is stored for transport: Corbicula; Scopa_leg=Tibial scopa; Abdominal scopa; Crop (ingested); Seasonality: Activity during vegetation period: Spring (AT: III – V, SA: IX – XI); Early summer (AT: V – VI, SA: XI – XII); Summer (AT: VI – VIII, SA: XII – II); Late summer (AT: IX, SA: III); Whole vegetation period (AT: III – X, SA: IX – IV); ITD: Inter-tegular distance (mm) = The shortest linear distance measured between wing tegulae across the dorsal thorax (Cane, 1987)

**Appendix - Figures**


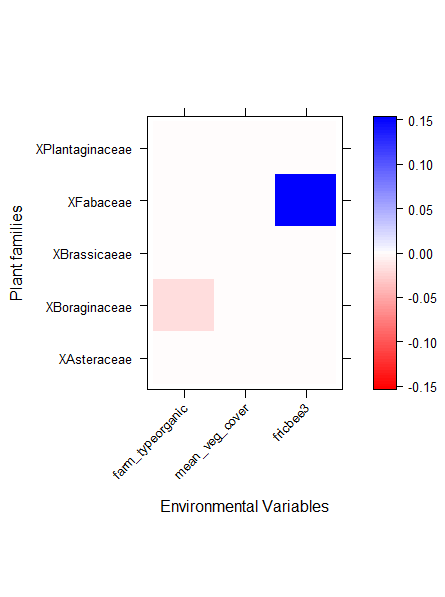

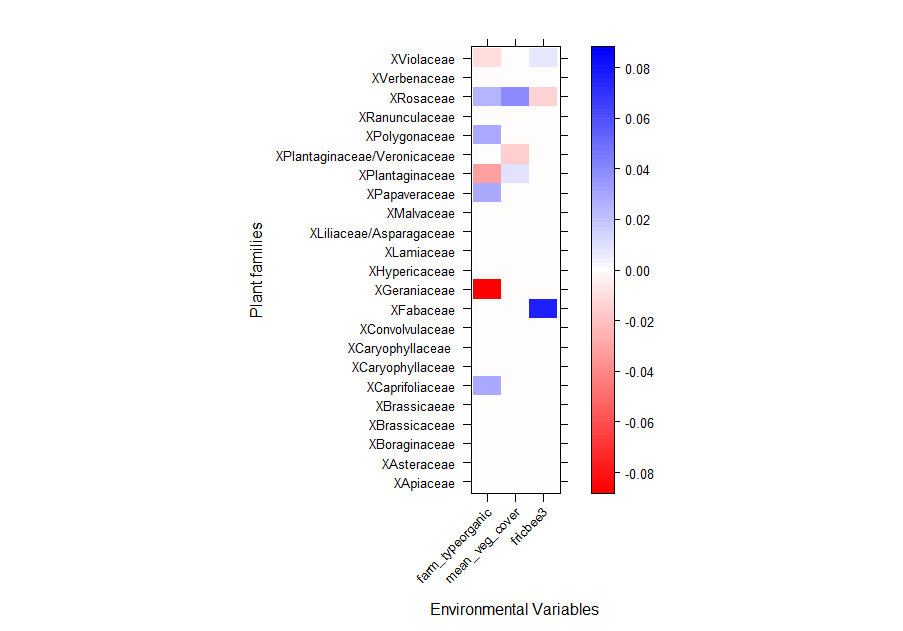


**a**

**b**

Fig S1: Effects of environmental variables on plant families flowering in vineyard inter-rows. (a) Austria and (b) South Africa. Abbreviations: mean_veg_cover: Mean vegetation cover per inter-row; fricbee3: Wild bee functional richness. Darker colours represent stronger correlations, with blue representing positive and red representing negative correlations.


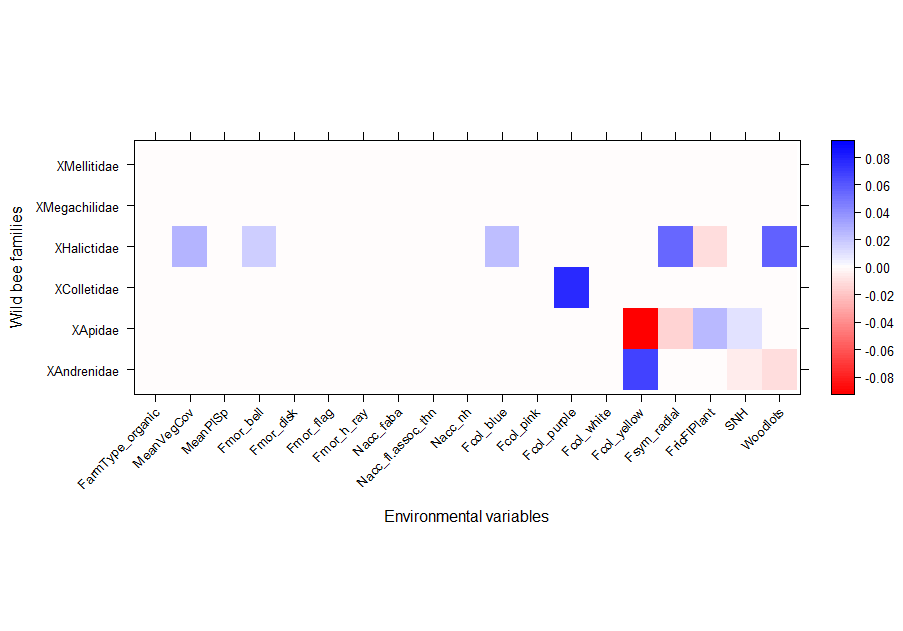

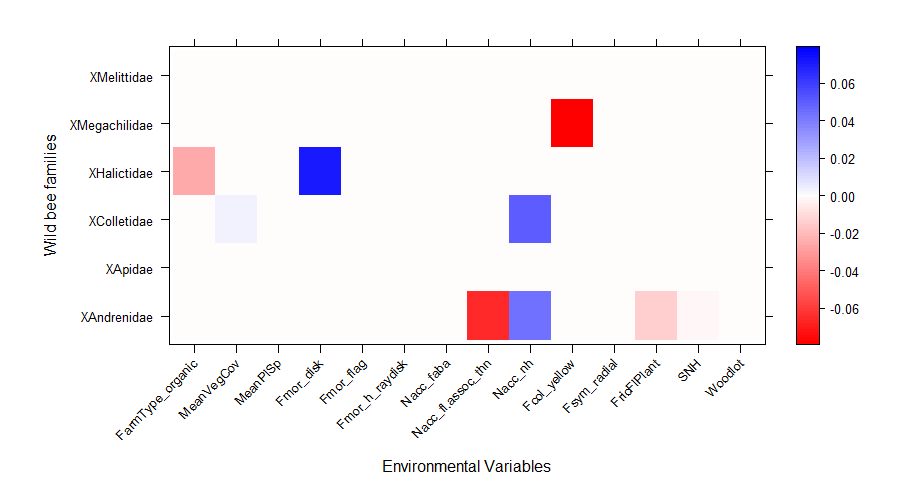


**a**

**b**

Fig S2: Effects of environmental variables on wild bee families in vineyard inter-rows. (a) Austria and (b) South Africa. Abbreviations: MeanVegCov: Mean vegetation cover per inter-row, MeanPlSp: Mean plant species richness per inter-row; Fmor: Flower morphology; bell: Bell & funnel flowers; disk: Disk flowers; flag: Flag blossom; h_ray: Asteraceae, only ray flower heads; h_raydisk: Asteraceae, ray & disc flower heads; Nacc: Nectar accessibility; faba: Fabaceae type; nh: Nectar ± hidden; fl.assoc_thn: Flower associations with totally hidden nectar; thn: Flowers with totally hidden nectar; Fsym: Flower symmentry; radial: Flowers with radial symmetry. Fcol: Flower colour; FricFlPlant: Flowering plant functional richness, SNH: Semi-natural habitats. Darker colours represent stronger correlations, with blue representing positive and red representing negative correlations.

**Appendix – TRY references:**

**Literature references of functional traits including detailed references from TRY request**

Bayer, E., Buttler, K.P., Finkenzeller, X. & Grau, J. (1987) Pflanzen des Mittelmeerraumes. Illustriert von J. Grau. Mosaik Verlag, München.

Briemle, G., Nitsche, S. & Nitsche, L. (2002) Nutzungswertzahlen für Gefäßpflanzen des Grünlandes. In: Klotz, S., Kühn, I. & Durka, W. [eds.]: BIOLFLOR - Eine Datenbank zu biologisch-ökologischen Merkmalen der Gefäßpflanzen in Deutschland. -Schriftenreihe für Vegetationskunde 38. Bundesamt für Naturschutz, Bonn.

Doohan, D.J. & Monaco, T.J. (1992) The biology of Canadian weeds. 99. Viola arvensis Murr. Canadian Journal of Plant Science, 72, 187–201.

Dressler, S., Schmidt, M. & Zizka, G. (2014) Introducing African Plants—A Photo Guide—An Interactive Photo Data-Base and Rapid Identification Tool for Continental Africa. Taxon, 63, 1159–1161.

Durka, W. (2002) Phylogenie der Farn- und Blütenpflanzen Deutschlands. - In: Klotz, S., Kühn, I. & Durka, W. [Hrsg.]: BIOLFLOR - Eine Datenbank zu biologisch-ökologischen Merkmalen der Gefäßpflanzen in Deutschland. - Schriftenreihe für Vegetationskunde 38: 75-91. Bundesamt für Naturschutz, Bonn.

Durka, W. (2002): Blüten- und Reproduktionsbiologie. In: Klotz, S., Kühn, I. & Durka, W. [eds.]: BIOLFLOR - Eine Datenbank zu biologisch-ökologischen Merkmalen der Gefäßpflanzen in Deutschland. - Schriftenreihe für Vegetationskunde 38: 133-175. Bundesamt für Naturschutz, Bonn.

Durka, W. (2002): Chromosomenzahlen, Ploidiestufen und DNA-Gehalte. In: Klotz, S., Kühn, I. & Durka, W. [eds.]: BIOLFLOR - Eine Datenbank zu biologisch-ökologischen Merkmalen der Gefäßpflanzen in Deutschland. - Schriftenreihe für Vegetationskunde 38. Bundesamt für Naturschutz, Bonn.

Green, W. (2009) USDA PLANTS Compilation , version 1, 09-02-02 [WWW Document]. National Plant Data Center: Baton Rouge, LA 70874-74490 USA. URL [accessed on 2009].

Haeupler, H. & Muer, T. (2000) Bildatlas der Farn- und Blütenpflanzen Deutschlands. Ulmer, Stuttgart.

Klotz, S. & Kühn, I. (2002): Blattmerkmale. - In: Klotz, S., Kühn, I. & Durka, W. [Hrsg.]: BIOLFLOR - Eine Datenbank zu biologisch-ökologischen Merkmalen der Gefäßpflanzen in Deutschland. - Schriftenreihe für Vegetationskunde 38: 119-126. Bundesamt für Naturschutz, Bonn.

Klotz, S. & Kühn, I. (2002): Indikatoren zum anthropogenen Einfluss auf die Vegetation. In: Klotz, S., Kühn, I. & Durka, W. [eds.]: BIOLFLOR - Eine Datenbank zu biologisch-ökologischen Merkmalen der Gefäßpflanzen in Deutschland. - Schriftenreihe für Vegetationskunde 38: 241-246. Bundesamt für Naturschutz, Bonn.

Klotz, S. & Kühn, I. (2002): Soziologische Bindung der Arten. In: Klotz, S., Kühn, I. & Durka, W. [eds.]: BIOLFLOR - Eine Datenbank zu biologisch-ökologischen Merkmalen der Gefäßpflanzen in Deutschland. - Schriftenreihe für Vegetationskunde 38: 273-281. Bundesamt für Naturschutz, Bonn.

Klotz, S. & Kühn, I. (2002): Ökologische Strategietypen. - In: Klotz, S., Kühn, I. & Durka, W. [Hrsg.]: BIOLFLOR - Eine Datenbank zu biologisch-ökologischen Merkmalen der Gefäßpflanzen in Deutschland. - Schriftenreihe für Vegetationskunde 38: 197-201. Bundesamt für Naturschutz, Bonn.

Klotz, S., Kühn, I. & Durka, W. [eds.]: BIOLFLOR - Eine Datenbank zu biologisch-ökologischen Merkmalen der Gefäßpflanzen in Deutschland. - Schriftenreihe für Vegetationskunde 38. Bundesamt für Naturschutz, Bonn.

Knuth, P. (1898) Handbuch der Blütenbiologie. Verlag von Wilhelm Engelmann, Leipzig.

Krumbiegel, A. (2002): Morphologie der vegetativen Organe (außer Blätter). In: Klotz, S., Kühn, I. & Durka, W. [eds.]: BIOLFLOR - Eine Datenbank zu biologisch-ökologischen Merkmalen der Gefäßpflanzen in Deutschland. - Schriftenreihe für Vegetationskunde 38: 93-118. Bundesamt für Naturschutz, Bonn.

Kubitzki, K., Rohwer, J.G. & Bittrich, V. (1993) Flowering Plants. Dicotyledones. Springer, Berlin.

Kugler, H. (1970) Blütenökologie. 2nd edn. Gustav Fischer Verlag, Stuttgart.

Kühn, I. Klotz, S. (2002): Angaben zu den Arealen. In: Klotz, S., Kühn, I. & Durka, W. [eds.]: BIOLFLOR - Eine Datenbank zu biologisch-ökologischen Merkmalen der Gefäßpflanzen in Deutschland. - Schriftenreihe für Vegetationskunde 38: 227-239. Bundesamt für Naturschutz, Bonn.

Kühn, I., Klotz, S. (2002): Floristischer Status und gebietsfremde Arten. In: Klotz, S., Kühn, I. & Durka, W. [eds.]: BIOLFLOR - Eine Datenbank zu biologisch-ökologischen Merkmalen der Gefäßpflanzen in Deutschland. - Schriftenreihe für Vegetationskunde 38: 47-56. Bundesamt für Naturschutz, Bonn.

Kühn, I., Durka, W. & Klotz, S. (2004) BiolFlor - A new plant-trait database as a tool for plant invasion ecology. Diversity and Distributions, 10, 363–365.

Melin, A., Krenn, H.W., Bowie, R.C.K., Beale, C.M., Manning, J.C. & Colville, J.F. (2019) The allometry of proboscis length in Melittidae (Hymenoptera: Apoidae) and an estimate of their foraging distance using museum collections. PLoS ONE, 14, 1–18.

Müller, H. (1881) Alpenblumen, ihre Befruchtung durch Insekten und ihre Anpassung an dieselben. Wilhelm Engelmann, Leipzig.

Otto, B. (2002): Merkmale von Samen, Früchten, generativen Germinulen und generativen Diasporen. In: Klotz, S., Kühn, I. & Durka, W. [eds.]: BIOLFLOR - Eine Datenbank zu biologisch-ökologischen Merkmalen der Gefäßpflanzen in Deutschland. - Schriftenreihe für Vegetationskunde 38. Bundesamt für Naturschutz, Bonn.

Schubert, R., Jäger, E.J. & Werner, K. (1987) Exkursionsflora für die Gebiete der DDR und BRD. Band 3, Atlas der Gefäßpflanzen. 1st edn. Volk und Wissen Verlag, Berlin.

Trefflich, A., Klotz, S. & Kühn, I. (2002): Blühphänologie. In: Klotz, S., Kühn, I. & Durka, W. [eds.]: BIOLFLOR - Eine Datenbank zu biologisch-ökologischen Merkmalen der Gefäßpflanzen in Deutschland. - Schriftenreihe für Vegetationskunde 38: 127-131. Bundesamt für Naturschutz, Bonn.

**Other references in Appendix:**

Cariveau, D.P., Nayak, G.K., Bartomeus, I., Zientek, J., Ascher, J.S., Gibbs, J., *et al.* (2016) The allometry of bee proboscis length and its uses in ecology. *PLoS ONE*, **11**, 1–13.

Kattge, J., Bönisch, G., Díaz, S., Lavorel, S., Prentice, I.C., Leadley, P., *et al.* (2020) TRY plant trait database – enhanced coverage and open access. *Global Change Biology*, **26**, 119–188.

Kendall, M.L. (2018) Package ‘ pollimetry .’

Kugler, H. (1970) *Blütenökologie*. 2nd edn. Gustav Fischer Verlag, Stuttgart.

Melin, A., Krenn, H.W., Bowie, R.C.K., Beale, C.M., Manning, J.C. & Colville, J.F. (2019) The allometry of proboscis length in Melittidae (Hymenoptera: Apoidae) and an estimate of their foraging distance using museum collections. *PLoS ONE*, **14**, 1–18.

Müller, H. (1881) *Alpenblumen, ihre Befruchtung durch Insekten und ihre Anpassung an dieselben*. Wilhelm Engelmann, Leipzig.
